# The Proto Type Galectin Drgal1-L2 from Zebrafish Hinders Infection by the Infectious Hematopoietic Necrosis Virus by Binding to Its Glycosylated Receptors on the Epithelial Cell Surface

**DOI:** 10.3390/biom16060882

**Published:** 2026-06-15

**Authors:** Kelsey Abernathy, Sheng Wang, Chiguang Feng, Justin Mancini, Guanghui Zong, Nuria González-Montalbán, Lai-Xi Wang, Gerardo R. Vasta

**Affiliations:** 1 Department of Microbiology and Immunology, University of Maryland School of Medicine, UMB, and Institute of Marine and Environmental Technology, Baltimore, MD 21202, USA; 2 State Key Laboratory for Biocontrol, School of Life Sciences, Sun Yat-sen University, Guangzhou 510275, China; 3 Department of Chemistry and Biochemistry, University of Maryland, College Park, MD 20742, USA; gzong@umd.edu (G.Z.);

**Keywords:** zebrafish galectin, epidermal mucus, viral receptors, viral envelope glycans, mucus glycans, anti-viral defense

## Abstract

Galectins are β-galactosyl-binding lectins with key roles in immune regulation and as pattern recognition receptors. To address their potential role(s) in viral infection of mucosal epithelia we currently investigate adhesion and entry mechanisms of the infectious hematopoietic necrosis virus (IHNV) using the zebrafish (*Danio rerio*) model system. We previously reported the recognition of IHNV envelope glycoprotein by the zebrafish galectin Drgal1-L2 and its inhibitory activity for viral adhesion to epithelial cells. Subsequently, we determined the structure of Drgal1-L2 and proposed a mechanism for Drgal1-mediated inhibition of IHNV spike fusion to the host epithelial cell. We now show that Drgal1 can also hinder viral adhesion and infection by binding to glycans on the host cell surface and epidermal mucus. We identified fibronectin, the reported IHNV receptor, as the cell surface glycoprotein recognized by Drgal1-L2. Surprisingly, IHNV also adhered in vitro to purified β1integrin, and pre-exposure of either IHNV or the immobilized β1integrin to Drgal1-L2 hindered IHNV adhesion. Binding of either anti-fibronectin or anti-β1integrin antibodies to the cell surface partially inhibited IHNV adherence. Drgal1-L2 also hindered IHNV adhesion by binding to mucus glycans. Taken together, our results suggest complementary mechanisms by which Drgal1-L2 may protect mucosal epithelial cells against IHNV infection and tentatively identify β1integrin as a novel receptor for IHNV.

## 1. Introduction

Protein–carbohydrate interactions are ubiquitous and essential to biological systems, both as cell adhesion molecules and as drivers and modulators of their physiology and homeostatic balance [1,2,3]. Among carbohydrate-binding proteins, lectins from viruses, bacteria, and parasites can facilitate their attachment and entry into the host [4,5,6], while reciprocally, lectins from both invertebrate and vertebrate hosts, can function as pattern recognition receptors (PRRs) by recognizing carbohydrates on the surface of the pathogen or parasite, and as effector factors promoting their clearance [7,8].

Within the hosts’ diverse lectin repertoire, galectins comprise a functionally diverse and taxonomically ubiquitous family of β-galactoside-binding lectins [9,10,11]. In vertebrates, galectins are currently classified into three major types, “proto”, “chimera”, and “tandem-repeat”, based on their subunit structure [12]. Proto- and chimera-type galectins contain a single carbohydrate-recognition domain (CRD) per subunit, while in the TR galectins, two CRDs are joined by a flexible linker peptide. Among the proto type, galectin-1 (Gal1), which can oligomerize into non-covalently bound dimers, is the best characterized example [11,13].

Galectins are expressed in the cytosol, and can be translocated into the nucleus, or released by an unconventional mechanism into the extracellular space, where they can bind and cross-link extracellular matrix and cell surface glycoproteins that display terminal galactose [11,13]. Thus, by binding to these “self” ligands, galectins can play key roles in development, tissue repair, cancer, pregnancy, immune homeostasis, obesity, and diabetes [14,15,16,17,18,19,20,21,22,23,24,25,26,27]. During recent years, however, a gradual paradigm shift has taken place through the realization that galectins can also bind to “non-self” glycans on the surface of potentially pathogenic viruses, bacteria, and eukaryotic parasites and function as PRRs [28,29,30,31,32,33] as well as carry out regulatory and effector functions aimed at enhancing the host immune response to neutralize and clear the infectious threat [34,35,36,37].

Along with the development of aquaculture of salmonid species such as trout and salmon in USA, Asia and Europe, the infectious hematopoietic necrosis virus (IHNV) has become an important fish pathogen, with mortality close to 100% in juvenile fish [38,39,40]. IHNV is an enveloped Novirhabdovirus (*Rhabdoviridae*) that enters the fish through epithelial cells at the mucosal interface with the external environment, including the skin, gills, nasal cavity, and gut [41,42,43]. Infection by IHNV involves interaction of the viral envelope glycoprotein spikes with the glycoprotein receptor, a truncated fibronectin on the fish epithelial cell surface, followed by fusion, internalization via the clathrin-mediated pathway [43,44]. The stressful conditions of high-density aquaculture, such as overcrowding and unsuitable environmental temperature, pH, and hypoxia can lead to IHNV outbreaks in both juvenile and adult fish [45,46]. However, the defense mechanisms responsible for preventing IHNV infections in low-stress environments under optimized nutritional and operational factors, are not fully understood.

In addition to innate and adaptive immune mechanisms that constitute the typical antiviral response, among the external defense strategies that prevent access of viruses to the cell surface in the fish mucosal epithelia, the first barrier at the interface with the environment is the continuous mucus film that coats the skin, gills, and gut. This viscous mucus film is regularly sloughed off and constitutes both a mechanical and microbicidal barrier that prevents pathogens from colonizing the epithelial surface [47,48,49,50,51,52,53,54]. Secretion of epidermal mucus, however, is significantly diminished in quantity/quality under the abovementioned stressful environmental conditions of high-density aquaculture, and furthermore, the frequent loss of integrity of the mucus film by mechanical trauma due to overcrowding enables pathogens to reach the epithelial surface and cause infection [55,56,57,58,59,60,61,62,63,64,65,66,67].

In prior studies we addressed the roles of galectins in IHNV infection using the zebrafish as a model system and reported that the zebrafish proto type galectin isoform Drgal1-L2 and the chimera type galectin Drgal3 are secreted into the epidermal mucus coat and by binding to *N*-acetyllactosamine (LacNAc; Galβ1,4GlcNAc) on the IHNV envelope they can inhibit viral adhesion to fish epithelial cells [68]. Further, our structural and in silico studies on Drgal1-L2 revealed the detailed interactions with oligosaccharides from the IHNV glycosylated envelope, and a potential mechanism for the direct inhibition of attachment and fusion of the envelope spikes to the host epithelial cell [69]. However, Drgal1-L2 can also bind to endogenous (“self”) glycans on the surface of fish epithelial cells [68], raising the possibility that it can also inhibit viral attachment by an alternative or synergistic mechanism consisting of Drgal1-L2 binding to the glycosylated receptors for IHNV on the epithelial cell surface. Furthermore, it is also possible that by binding to zebrafish epidermal mucus glycans, Drgal1-L2 could hinder viral access to cell surface receptor(s) by crosslinking virions to mucus.

Thus, to gain insight into the abovementioned potential alternative or synergistic defense mechanisms mediated by Drgal1-L2 binding to “self” epithelial cell surface or epidermal mucus ligands, in the study reported herein we characterized the expression and localization of Drgal1-L2 in fish epithelial cell lines, identified its potential glycan ligands on the cell surface and epidermal mucus, and analyzed the capacity of the Drgal1-L2-ligand interactions to hinder viral adhesion to cell surface receptors. Our studies revealed that: (a) the binding of Drgal1-L2 to the fish epithelial cell surface not only hinders IHNV adhesion but significantly hinders infection; (b) in addition to adhering in vitro to purified fibronectin, the reported IHNV cell surface receptor [44], IHNV also adhered in vitro to purified β1integrin; (c) pre-exposure of either IHNV or the immobilized glycoproteins (fibronectin or β1integrin) to Drgal1-L2 specifically hindered IHNV adhesion in vitro, tentatively identifying β1integrin as a novel receptor for IHNV; (d) binding of either anti-fibronectin or anti-β1integrin antibodies to the cell surface partially inhibited IHNV adherence; and (e) binding of Drgal1-L2 to mucus glycans also hindered IHNV adhesion to epithelial cells. Taken together, our results revealed that in addition to hindering IHNV adhesion by binding to the virion envelope glycoproteins, the binding of Drgal1-L2 to cell surface and mucus glycans may equally hinder IHNV adhesion in vitro. Thus, the results from our study suggest that Drgal1-L2 may protect mucosal epithelial cells against IHNV infection by alternative or complementary mechanisms.

## 2. Material & Methods

### 2.1. Reagents

Tricaine mesylate (MS-222), chloramphenicol, kanamycin, and β-mercaptoethanol (BME), neuraminidase, β-galactosidase were purchased from Sigma-Aldrich (St. Louis, MO, USA). PNGase F was purchased from New England Bio labs (Ipswich, MA, USA). EZ-Link™Sulfo-NHS-SS-Biotin kit, Protein A-Sepharose (Thermo Fisher Scientific, Waltham, MA, USA), DAPI stain custom PCR primers, DAPI stain, Oligo (dT), RevertAid RT, RiboLock (RI), DreamTaq master mix, and dNTP mix were purchased from Thermo Scientific (Waltham, MA, USA). DNase, Nuclease free H_2_O, 10 X cDNA mix, and EDTA were purchased from Invitrogen (Carlsbad, CA, USA). HRP-conjugated streptavidin and Alexa 488 conjugated streptavidin were purchased from Pierce (Thermo Fisher Scientific). The pET28b (+) vectors and Rosetta (DE3) pLysS competent cells, 25 U/mL Benzonase Nuclease, and lysozyme were obtained from Novagen (Madison, WI, USA). Cell culture media HBSS Ca/Mg and L-15 Leibovitz were purchased from Gibco (Gaithersburg, MD, USA). Trizol was obtained from Fisher Scientific (Pittsburgh, PA, USA). SYBR Green ROX qPCR Master mix was purchased from Qiagen, Venlo, The Netherlands. 1 X Protease Inhibitor Cocktail Set 1 was purchased from Calbiochem (San Diego, CA, USA). TMB substrate was purchased from SeraCare (Gaithersburg, MD, USA). Bovine serum fibronectin and anti-fibronectin antibody were purchased from Sigma Aldrich. Recombinant β1integrin and CD147 were purchased from Sino Biological (Chesterbrook, PA, USA). The anti-β1integrin antibody was purchased from GenTex (Irvine, CA, USA) and the anti-CD147 antibody from Sigma-Aldrich (St. Louis, MO, USA). Western Lightening Plus-ECL reagent, horseradish peroxidase (HRP), and carbohydrates used in binding-inhibition assays were from Sigma (St. Louis, MO, USA). Eight-chamber slides were purchased from Lab-Tek II (Santa Cruz, CA, USA). All other reagents were of the highest grade commercially available.

### 2.2. Animals, Collection of Epidermal Mucus and Tissues, and Preparation of Tissue Extracts

Zebrafish epidermal mucus was collected from animals that had been anesthetized and euthanized at the Aquaculture Research Center in our institution (IMET) under IACUC-approved protocols, and according to the standard methods previously described [68,70]. Briefly, wild-type zebrafish (30–40 fish, 0.45–0.65 g) were euthanized with MS-222 (Sigma Aldrich) in dechlorinated tap water at a lethal concentration according to the protocol provided by the manufacturer. The euthanized fish were placed in a Petri dish on ice and mucus was collected from the dorsal and lateral surfaces of the fish by gently pressing a scalpel in a downward motion across the scales forward and back. Care was taken to not break the skin to avoid blood contamination of the mucus sample. Mucus from 5 to 10 fish was pooled to yield 200–500 µL of mucus with an average protein concentration of 5–8 mg/mL (1–2 mg/mL mucus/fish). Mucus pools were diluted in 50% volume of PBS 1X with Protease Inhibitor Cocktail Set I (PICSI; EMD Millipore Corp, Merck KGaA, Darmstadt, Germany). Samples were vortexed vigorously at 30 s intervals and stored at −20 °C. Samples were quantified for protein concentration using Bio-Rad Protein Assay Kit (Bio-Rad Laboratories, Hercules, CA, USA).

### 2.3. Cell Culture

The epithelial cell line (EPC; *epithelioma papulosum cyprini* cell line; CRL-2872, ATCC) from the fathead minnow (*Pimephales promelas*) was a generous gift from Dr. Vikrham Vakharia, University of Maryland Baltimore County. The liver cell line (ZFL; CRL-2643, ATCC) from zebrafish (*Danio rerio*) was a generous gift from Dr. Rosemary Jagus, University of Maryland Center for Environmental Sciences. EPC cells were grown in minimal essential medium (MEM; Cellgro, Lincoln, NE, USA) supplemented with 10% fetal bovine serum (FBS; Quality Biological, Gaithersburg, MD, USA), 2 mM L-glutamine and penicillin-streptomycin at 26 °C. ZFL cells were grown in Leibovitz’s L-15 medium supplemented with penicillin-streptomycin at 28 °C. Both cell lines were cultured in an atmosphere of 100% air with humidification according to our standard protocol [68].

### 2.4. Virus Propagation, Purification, and Labeling

The recombinant IHNV strain 220-90 carrying enhanced green fluorescent protein (eGFP) was propagated by multiple passages on confluent layers of EPC cells, as we reported elsewhere [68]. In brief, confluent EPC cells grown in 75 cm^2^ flasks at 26 °C were infected at a multiplicity of infection (MOI) of 0.1 in the presence of MEM and 2% FBS. The infection was allowed to proceed at 16 °C until extensive cytopathic effects were observed. The supernatant was harvested 3–4 days post-infection (dpi), clarified by low-speed centrifugation, then separated on a discontinuous cesium chloride (CsCl) gradient by ultracentrifugation at 28,000× *g*. The banded virus was resuspended in TEN buffer (1 mM EDTA, 10 mM Tris-HCl, 1 M NaCl, pH 7.4) and stored at −80 °C. Virus titers were measured by endpoint dilution on EPC cells [71]. For biotinylation, the 1X TEN buffer was exchanged with phosphate-buffered saline (PBS) by overnight dialysis at 4–8 °C prior to the purified recombinant virus being reacted with 1 mg/mL EZ-Link Sulfo-NHS-Biotin (Pierce) in accordance with the manufacturer’s recommendations. Briefly, virus (5 mg/mL) were incubated with 10 mM of biotin reagent for 2 h on ice, followed by buffer exchange to remove the non-reacted reagent.

### 2.5. Production and Purification of Antibodies Against IHNV Virions

IHNV virions (5 mg total inoculum) purified from cell cultures as described above were provided to Proteintech Group, Inc. (Rosemont, IL, USA) for the immunization of New Zealand White rabbits. Antiserum titers against IHNV were periodically monitored by enzyme-linked immunosorbent assay (ELISA) as previously described [68]. Purification of immunoglobulins was carried out with Protein A-Sepharose and specificity tested by Western blot by comparing side-by-side binding patterns to the purified IHNV virions and extracts of IHNV-infected and uninfected EPC cells, as previously described [68]. Concentration of the purified immunoglobulins was determined by protein concentration on Nanodrop 2000 Spectrophotometer (Thermo Scientific, Thermo Fisher Scientific Inc., Waltham, MA, USA).

### 2.6. IHNV Plaque Assay

Monolayers of EPC cells grown in 24-well flat-bottom plates to 90% confluency were incubated for 2 h with doubling dilutions of recombinant Drgal1-L2 (5, 25, or 50 µg/mL) in HBSS Ca/Mg (Gibco), or with HBSS alone. Subsequently, the monolayers were washed twice with HBSS, followed by inoculation with increasing PFU of purified IHNV virions and incubation for 1 h at 16 °C. Next, the cells were washed three times with HBSS to remove any unbound virus, and infections proceeded for 5–7 days under 0.75% methyl cellulose overlay at 16 °C. Cells were fixed and stained with a 1% crystal violet solution. The stain in each well was eluted with acetic acid (33%), 200 µL of the supernatant were transferred into 96 well plates, and the absorbance was measured at 590 nm.

### 2.7. Experimental Infections with IHNV

For experimental infections, EPC cells were plated on 6-well plates (5 × 10^6^ PFU/well) one day before experiment for attachment. After the culture supernatant was removed, 1 mL of IHNV (5 × 10^6^ PFU/mL) or culture medium only (negative control) was applied onto each well and incubated for 1 h at 4 °C, followed by a 24 h incubation at 16 °C. The infected cells were harvested at the end point for processing and further analysis.

### 2.8. Expression of Drgal1 Isoforms by EPC and ZFL Cell Lines

Expression of Drgal1 isoforms by the EPC and ZFL cell lines was analyzed by quantitative real-time polymerase chain reaction (qRT-PCR) as previously described [68,72]. Briefly, total RNA from zebrafish tissues was treated with DNase I (Invitrogen) and reverse transcribed into cDNA using the Reverse Transcriptase System (Promega, Madison, WI, USA) following the manufacturer’s protocol with oligo dT primers. For quantitative PCR, 1 µL template of cDNA was mixed with 10 µL SYBR Green ROX qPCR Master mix (Qiagen), 8.2 µL of H_2_O and 0.4 µL of the oligonucleotide primers sets (Sigma-Aldrich) designed based on reference genome coding sequences of the zebrafish Drgal1 isoforms Drgal1-L1 (*lgals1l1*), -L2 (*lgals1l2*), and -L3 (*lgals1l3*) [68,72]. Primer sequences used are as follows: Drgal1-L1 (From GenBank accession number AW174841) forward: 5′-CGCGGAATGTTCGTGATG-3′; Drgal1-L1 reverse: 5′-CCCTTGGATCCTAGCTTGGC-3′; Drgal1-L2 forward: 5′-CCAGTGCACTATAGTGTGCAATTC-3′; Drgal1-L2 reverse: 5′-TCATTGGTGAATGTGATTTTTATCT-3′; Drgal1-L3 forward: 5′-GCAGCTCCACCAACAACTCAG-3′; Drgal1-L3 reverse: 5′-CGTGTGTGAAGGCATCGTCT-3′. Amplification was carried out on a Fast 7500 Real-Time PCR System (Applied Biosystems, Foster City, CA, USA), with 40 cycles of 95 °C for 15 s, 62 °C for 1 min, and 70 °C for 1 s. Fluorescence measurements were taken at 70 °C for 1 s during each cycle. Relative expression level of each gene was calculated using Liyak (2^−ΔΔCt^) method after normalization to endogenous β-actin.

### 2.9. Glycotype Profiles of EPC and ZFL Cells

The surface glycosylation of EPC and ZFL cells was visualized by the binding of labeled plant lectins of well-established specificity. For this, EPC cells (5 × 10^6^) and ZFL cells (5 × 10^6^) were fixed with 4% PFA and subsequently stained with 10 µg/mL FITC-conjugated plant lectins in the presence of 1% BSA for 1 h at room temperature (RT). The plant lectins used are as follows: Concanavalin A (ConA; specific for Manα1,6/3Man; tested at 2.5 mg/mL); *Maackia amurensis* II agglutinin (MAA II; Neu5Acα2,3Galβ1,3GalNAc; 5 mg/mL); peanut agglutinin (PNA; Galβ1,3GalNAc; 5 mg/mL); soybean agglutinin (SBA, GalNAcβ1,4Gal; 5 mg/mL); *Sambucus nigra* agglutinin (SNA; Neu5Acα2,6Gal/GalNAc; 5 mg/mL); *Ulex europaeus* agglutinin (UEA; Fucα1,2Galβ1,4GlcNAc; 5 mg/mL); and wheat germ agglutinin (WGA; GlcNAcβ1,4GlcNAc; 2.5 mg/mL). After three washes in PBS, the cells were suspended at 10^7^ cells/mL in PBS and analyzed in an BD Accuri C6 flow cytometer (BD Biosciences, Milpitas, CA, USA) as previously described [73]. Mean fluorescence intensity (MFI) of 100,000 gated events (95.5–98.7% of total) was collected for each sample.

### 2.10. Glycosidase Treatment of Glycoproteins

Bovine serum fibronectin (FN) (Sigma Aldrich) was treated with glycosidases to remove specific sugars. To cleave sialyated glycans bound to FN, 500 µg of the glycoprotein was incubated with 3 U of neuraminidase from *Clostridium perfringens* (Sigma Aldrich) overnight at 37 °C. The sample was then dialyzed in PBS to remove cleaved sialic acid. This sample was designated nFN. To cleave β-galactose from FN, 250 µg of nFN were incubated with 5 U of β-galactosidase from *E. coli* (Sigma Aldrich) overnight at 37 °C. The sample was then dialyzed in PBS to remove cleaved galactose. The product was designated n/gFN. To cleave N-linked glycans, 500 µg of FN was mixed with denaturing buffer (New England Bio Labs) and heated at 100 °C for 10 min, followed by addition of G7 buffer (New England Bio Labs) and NP40 as recommended by the manufacturer, incubation with 3 U of PNGase F (New England Bio Labs) overnight at 37 °C, and then it was dialyzed in PBS to remove cleaved glycans. The product was designated pFN. The same treatment with PNGase F was repeated with β1integrin, with the resulting product designated as pβ1integrin.

### 2.11. Glycotype of Recombinant β1integrin

The glycosylation of the recombinant β1integrin and bovine FN, used as a control, was visualized by the binding of labeled plant lectins of well-established specificity. For this, the lyophilized β1integrin (50 µg) (Expressed in CHO cells; Sino Biological) was reconstituted in PBS pH 7.4 according to the manufacturer’s instructions for a final concentration of 200 µg/mL. A bovine fibronectin (Sigma Aldrich) solution was also prepared at a concentration of 200 µg/mL. The glycoprotein solutions were mixed with loading buffer, heated, clarified by centrifugation, resolved in 12-well, 4–15% gradient SDS-PAGE gels (Qiagen), and transferred to polyvinylidene difluoride (PVDF) membranes. The membranes were blocked with 3% BSA in PBS overnight with shaking, washed, and incubated with the biotinylated plant lectins for 2 h at RT. The membranes were interrogated with the following lectins: *Maackia amurensis* agglutinin I (MAA I; specific for Neu5Acα2-3Galβ1-4GlcNAc/Glc, tested at 2.5 mg/mL), *Erythrina cristagalli* agglutinin (ECA; Galβ1-4GlcNAc, 2.5 mg/mL); peanut agglutinin (PNA; Galβ1-3GalNAc, 5 mg/mL); *M. amurensis* agglutinin II (MAA II; Neu5Acα2-3Galβ1-3GalNAc, 5 mg/mL); and *Sambucus nigra* agglutinin (SNA; Neu5Acα2-6Gal/GalNAc, 5 mg/mL). After incubation with the biotinylated plant lectins, streptavidin-HRP was added, and the membranes incubated for 1 h at RT. The membranes were washed and developed by chemiluminescence with a 10 s exposure, and the relative intensity of the bands was scored with a numerical value within an arbitrary scale from 1 to 5.

### 2.12. Expression and Purification of Recombinant Drgal1-L2

Expression and purification of recombinant Drgal1-L2 (rDrgal1-L2) was carried out as previously described [68]. Briefly, the histidine-tagged Drgal1-L2 (Genbank accession no. AY421704) in pET28b (+) expression vector was transformed into *Escherichia coli* Rosetta (DE3) pLysS competent cells (Novagen). Plasmid-carrying cells were grown in Luria–Bertani (LB) broth with antibiotics (Chloramphenicol, 34 mg/mL; kanamycin, 15 mg/mL) and recombinant protein expression was induced with 0.1 mM isopropyl D-thiogalactoside (IPTG) for 16 h at 23 °C with shaking. The soluble protein fraction was extracted from the cell pellet with BugBuster Protein Extraction Reagent (Novagen) containing 1 mM phenylmethylsulfonyl fluoride (PMSF), 1X Protease Inhibitor Cocktail Set I (Calbiochem), 20 mg/mL lysozyme, 25 U/mL Benzonase Nuclease (Novagen) and 0.07% β-mercaptoethanol (BME) and centrifuged at 14,000× *g* at 4 °C for 15 min. The clarified supernatant was loaded onto a pre-equilibrated chromatography column packed with 4 mL of divinyl sulfone-conjugated lactosyl-Sepharose slurry, and washed with wash buffer (1:10 PBS, 0.07% BME), then the purified rDrgal1-L2 was eluted with elution buffer (1:10 PBS, 0.07% BME, 100 mM lactose). Glycerol was added to a final concentration of 50% (*v*/*v*) and stored at −20 °C. In some experiments, rDrgal1-L2 was biotinylated as previously described [68].

### 2.13. Production of Anti-Drgal1-L2 Antibodies

Anti-Drgal1 antiserum was prepared in New Zealand White rabbits at Duncroft (Lovetsville, VA, USA) by multiple subcutaneous and intramuscular injections of affinity purified Drgal1 (100 µg/injection). Antiserum titers against Drgal1 were periodically monitored by enzyme-linked immunosorbent assay (ELISA) as previously described [68]. Immunoglobulins were purified on Protein A-Sepharose and confirmed for specificity in Western blot by comparing side-by-side binding patterns to the affinity-purified Drgal1 and zebrafish crude tissue extracts as previously described [68]. Anti-Drgal1-L2 antiserum was prepared in New Zealand White rabbits at Duncroft (Lovetsville, VA, USA), following a similar protocol as previously described [72]. Monitoring of the antiserum titers and specificity against rDrgal1-L2 and purification of immunoglobulins was carried out as reported elsewhere [68,72].

### 2.14. Surface Plasmon Resonance (SPR) Analysis of Drgal1-L2 Binding to Glycoproteins

SPR measurements were performed on a Biacore T200 instrument (GE Healthcare, Chicago, IL, USA) at 25 °C. Approximately 2000 resonance units (RU) of fibronectin, β1integrin, asialofetuin, and BSA were immobilized on a CM5 sensor chip in a sodium acetate buffer (50 μg/mL, pH 4.0), using the amine coupling kit provided by the manufacturer. A reference channel was immobilized with ethanolamine. Binding analyses were performed by injecting a solution of rDrgal1-L2 over four cells at 2-fold increasing concentration in HBS-P running buffer (10 mM HEPES, 150 mM NaCl, P20 surfactant 0.05% *v*/*v*, pH 7.4) containing 10 mM BME at a flow rate of 20 μL/min for 2 min and allowed to dissociate for another 5 min. The surface was regenerated after each cycle by injecting a 2 M MgCl_2_ water solution for 3 min at a flow rate of 30 μL/min. Kinetic analyses of the experimental data to obtain the equilibrium dissociation constant (*K*_D_) were performed using two different models (the 1:1 Langmuir model and the two-state model) with the Biacore T200 Evaluation Software version 2.0 (GE Healthcare).

### 2.15. Expression of Fibronectin and β1integrin by EPC Cells

The expression of fibronectin and β1integrin by EPC cells and their display on the cell surface was analyzed by the detection of cross-reactive proteins in cell surface protein extracts by Western blot, and in intact cells by immunofluorescence histochemistry, both using the specific anti-fibronectin and anti-β1integrin specific antibodies, respectively.

### 2.16. Isolation of Cell Surface Proteins from EPC Cells

Biotinylation of the EPC cell surface, and subsequent isolation was done in accordance with the instructions from the Pierce Cell Surface Protein Isolation Kit (Thermo Scientific). Cells were grown to 90–95% confluency and biotinylated using Sulfo-NHS-SS-Biotin (Thermo Scientific) before being lysed and run through a NeutrAvidin Agarose Column (Thermo Scientific) to isolate the biotinylated cell surface proteins. Protein concentration was measured on a Nanodrop 2000 Spectrophotometer.

### 2.17. Detection of Fibronectin and β1integrin on Isolated Cell Surface Proteins from EPC Cells

Isolated EPC cell surface proteins were run on a 4–15% gradient SDS-PAGE Gel (SMOBIO, Hsinchu, Taiwan) and transferred to a PVDF membrane. The membranes were blocked with 5% non-fat dry milk for 1 h at RT and incubated with a 1:1000 dilution of purified polyclonal rabbit immunoglobulins specific for fibronectin or β1integrin (Sino Biological) overnight at 4 °C, followed by incubation with a 1:1000 dilution of HRP-conjugated anti-rabbit IgG (Invitrogen) at 1:4000. Detection was carried out by chemiluminescence using Western Lightning Plus-ECL reagent (Perkin Elmer, Waltham, MA, USA) [74].

### 2.18. Immunofluorescence Histochemistry

EPC cells were plated at 2 × 10^4^ cells/well in 200 μL of medium for each chamber slide (Lab Tek II, Thermo Scientific, Waltham, MA, USA) and grown to 60–70% confluency. Cells were washed, fixed with 4% paraformaldehyde (Thermo Scientific) for 10 min, and blocked with 3% BSA for 1 h at RT. The cells were then incubated overnight at 4 °C with 200 mL of a 1:500 dilution of either anti-Drgal1-L2, anti-fibronectin (Sigma Aldrich), anti-β1integrin (GeneTex, Irvine, CA, USA), or the appropriate combinations of these for the co-localization experiments. The cells were washed with PBS three times and then incubated with secondary antibody, Alexa 555-, FITC-, and/or TRITC-conjugated anti-rabbit (Life Technologies, Thermo Fisher Scientific, Waltham, MA, USA) at 1:200 dilution for 1 h at RT. After the cells were washed to remove excess antibody, they were incubated with DAPI stain (Thermo Scientific) at 1:1000 dilution and mounted in ProLong Antifade mounting medium (Thermo Fisher). Cells were imaged using the Revolve by Echo on EPI-Fluorescence setting for DAPI, Alexa 555, FITC, and TRITC respectively.

### 2.19. Solid Phase Assays to Test Drgal1-L2 Binding to Glycoproteins, IHNV, and EPC Cells

All experiments were carried out in triplicate and repeated at least twice. Solid phase binding assays were generally carried out as described elsewhere [73].

### 2.20. Binding of Drgal1-L2 to Glycoproteins

Each well of a 96-well microtiter plate was coated with 100 µL of 5 µg/mL of either fibronectin (Sigma Aldrich), β1integrin (Sino Biological), or CD147 (Sino Biological) and incubated for 2 h at 37 °C. The plate was blocked with 3% BSA overnight at 4 °C. The plate was then incubated with either increasing concentrations of biotinylated Drgal1-L2 (0–5 µg/mL). Binding was directly detected with HRP conjugated streptavidin (Pierce) at a 1:1000 dilution. The plate was developed using TMB substrate and the reaction was stopped by adding 1 M HCl. Absorbance values were read at 450 nm.

### 2.21. Binding of Drgal1-L2 to EPC Cells

Each well of a 96-well microtiter plate was seeded with 5 × 10^4^ EPC cells and incubated overnight. The plate was blocked with 3% BSA in HBSS CaMg (Gibco) overnight at 4 °C. The plate was then incubated with increasing concentrations of biotinylated Drgal1-L2 that had been preincubated with either PBS or 100 mM lactose as a specificity control. Galectin binding to EPC cells was directly detected with HRP conjugated streptavidin (Pierce) at a 1:1000 dilution. The plate was developed using TMB substrate (SeraCare) and the reaction was stopped by adding 1 M HCl. Absorbance values were read at 450 nm.

### 2.22. Binding of Drgal1-L2 to Immobilized IHNV

Each well of a 96-well microtiter plate coated with 100 µL of non-biotinylated IHNV at various dilutions (1.25 µg/mL, 2.5 µg/mL, and 5 µg/mL) for 2 h at 37 °C. The plate was blocked with 3% BSA overnight at 4 °C, then incubated with 10 µg/mL of biotinylated rDrgal1-L2 that had been pre-incubated with either PBS 1X or with 100 mM lactose for 1 h at RT. Binding was directly detected with HRP conjugated streptavidin (Pierce) at a 1:1000 dilution. The plate was developed using TMB substrate and the reaction was stopped by adding 1 M HCl. Absorbance values were read at 450 nm.

### 2.23. Solid Phase Assay for IHNV Adhesion and Adhesion-Inhibition to Glycoproteins and EPC Cells

Solid phase IHNV adhesion and adhesion-inhibition assays were generally carried out as we described elsewhere [68]. For binding-inhibition assays, the 50% inhibition concentration (IC50) was calculated (Prism Version 6) as 50 percent binding activity relative to the no-inhibitor control (100%; no inhibitor added).

### 2.24. Adhesion of IHNV to Immobilized Glycoproteins and Epidermal Mucus

Adhesion of IHNV to immobilized glycoproteins or zebrafish epidermal mucus was tested by a solid phase assay in which each well of a 96-well microtiter plate was coated with 100 µL of 5 µg/mL of either fibronectin (Sigma Aldrich), β1integrin (Sino Biological), CD147 (Sino Biological), or epidermal mucus (100 µg protein/mL) and incubated for 2 h at 37 °C. The plate was blocked with 3% BSA in HBSS CaMg (Gibco) overnight at 4 °C, and biotinylated IHNV that had been pre-incubated with Drgal1-L2 (30 µg/mL) or specific antibodies, or PBS (control) was added. Viral attachment to the immobilized glycoproteins or zebrafish epidermal mucus was detected directly with HRP conjugated streptavidin (Pierce) at a 1:1000 dilution. The plate was developed using TMB substrate (SeraCare) and the reaction was stopped by adding 1 M HCl. Absorbance values were read at 450 nm.

### 2.25. Adhesion of IHNV to EPC Cell Monolayers

To assess IHNV adhesion to EPC cells, each well of a 96-well microtiter plate was seeded with 5 × 10^4^ EPC cells, incubated overnight to 100% confluence, and incubated with biotinylated IHNV (MOI of 3 or 6) that had been pre-incubated with Drgal1-L2 (30 µg/mL) or PBS for 30 min, followed by three washes to remove the unbound virus. The 30 min time point was selected based on the reported period at which viral adhesion is optimal, without any viral replication taking place [75]. Viral attachment to EPC cells was directly detected with HRP conjugated streptavidin (Pierce) at a 1:1000 dilution. The plate was developed using TMB substrate (SeraCare) and the reaction was stopped by adding 1 M HCl. Absorbance values were read at 450 nm. Inhibition of IHNV adhesion by pre-treatment with Drgal1-L2 or specific antibodies was carried out as above for viral adhesion of glycoproteins and epidermal mucus.

### 2.26. Statistical Analyses

The mRNA and protein expression levels were quantified using Image J software (version 1.54t) to estimate the relative band intensities. Comparison of two groups was performed by Student’s T-test for the comparison of non-paired samples. All results with *p* < 0.05 were considered statistically significant.

## 3. Results

Based on our prior observations that: (1) the binding of the zebrafish proto-type galectin to the IHNV glycosylated envelope can hinder adhesion to fish epithelial cells, and (2) Drgal1 can also bind to the cell surface in a glycans dose-dependent and carbohydrate-specific manner [68], we hypothesized that Drgal1 secreted by the epithelial cells into the mucus could also hinder viral adhesion by binding to the reported cell surface IHNV receptor, a truncated FN, and block access of the virus to the epithelial cell surface. Furthermore, as other cell surface glycoproteins have been reported as galectin ligands, some of which have been reported as receptors for other viruses, we examined the possibility that the binding of Drgal1 to these surface receptors could also interfere with IHNV adhesion and infection. We first tested this hypothesis in an in vitro experimental format in which the key variables could be rigorously controlled, by using the fish cell lines ZFL, established from zebrafish liver, and EPC, an epithelial cell line from the fathead minnow. Finally, we investigated the potential synergistic defensive role of epidermal mucus in the abovementioned Drgal1 inhibitory activity for viral adhesion.

### 3.1. Expression of Drgal1 Isoforms in Fish Epithelial Cell Lines

**Figure 1:**
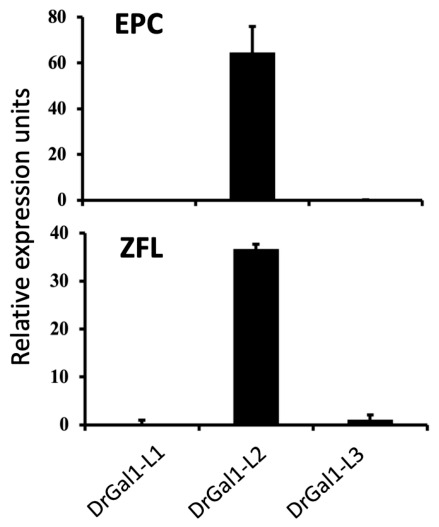
Expression Drgal1 Isoforms in the Fish Cell Lines EPC and ZFL. Transcription of Drgal1 isoforms (Drgal1-L1,2,3) by EPC and ZFL cell lines was analyzed by qRT-PCR as described in Materials and Methods. Total RNA was extracted from EPC (**top panel**) or ZFL cells (**bottom panel**), reverse transcribed to cDNA template, then amplified with the oligonucleotide primers sets described in Materials and Methods. Relative expression of each isoform (Drgal1-L1, Drgal1-L2, Drgal1-L3) over β-actin were calculated. Representative data of average of triplicate samples from three independent experiments are shown.

In a prior study we had identified three main isoforms of Drgal1 (Drgal1-L1, -L2 and -L3) that were differentially expressed and localized in different zebrafish tissues [70]. Thus, we first addressed the question if any particular Drgal1 isoform was preferentially expressed in the fish cell lines EPC and ZFL. The results of RNA levels from qRT-PCR showed that while the isoform Drgal1-L2 is expressed by both cell lines, the Drgal1-L3 isoform is only marginally expressed in the ZFL cell line, and Drgal1-L1 is not expressed by either cell type (Figure 1).

### 3.2. Glycotype of the Surface of the Epidermal Cell Lines EPC and ZFL

**Figure 2:**
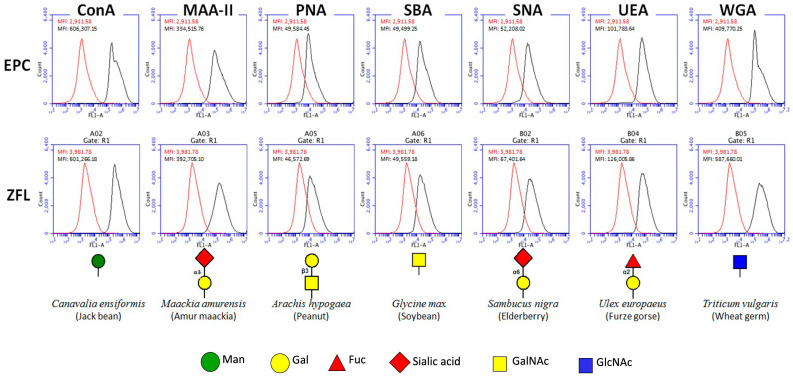
Glycotype Profiles of EPC and ZFL Cells: 5 × 10^6^ EPC cells (**top row**) or ZFL cells (**bottom row**) were fixed with 4% PFA and subsequently stained with 10 µg/mL FITC-conjugated plant lectins (red line) or blank control (black line) in the presence of 1% BSA. Mean fluorescence intensity (MFI) of 100,000 gated events (95.5–98.7% of total) collected for each sample was also shown. Representative data from three independent experiments are shown. The preferred oligosaccharide ligands for each lectin (and their sources) are shown below with the standard symbols. The displacement of the black lines (experimentals) to the right relative to the red lines (controls) indicated lectin binding to the oligosaccharide structures illustrated below, revealing similar glycosylation profiles on the surface of both EPC and ZFL cells.

Next, to rationalize our prior study’s observation regarding the binding of Drgal1-L2 to the epithelial cell surface, we addressed the question if the EPC and ZFL cells display the surface carbohydrate moieties that are typical ligands for Drgal1, such as LacNAc and lactose (Galβ1,4Glc). For this, we analyzed the glycotype of the surface of the epidermal cell lines EPC and ZFL using a panel of plant lectins of well-established specificity. The results showed that the surface of both epidermal cell lines EPC and ZFL displayed virtually identical glycans, with abundant non-reducing terminal sialic acid, galactose, GalNAc, and mannose (Figure 2). Importantly, the presence of galactosylated moieties suggest that these likely represent the structures that can be recognized by Drgal1-L2 on the cell surface of both cell lines. Based on the abovementioned results of similar expression of galectins (Figure 1) and glycotypes (Figure 2) of ZFL and EPC cells, from here on we selected the EPC cell line to carry out all further experiments. The rationale for the selection of EPC over the ZFL was further supported by the following observations: (a) EPC is an epithelial cell line, and therefore, representative of the skin and other mucosal epithelial cells to which IHNV will attach to; (b) IHNV can attach to and infect equally both EPC and ZFL, but replicates effectively and only forms lysis plaques in EPC, making this cell line advantageous for studies on IHNV infectivity and pathogenesis in fish; and (c) galectin sequences cloned from EPC are highly similar to those from ZFL and adult zebrafish, also supported by strong cross-reactivity of anti-zebrafish galectin antibodies as we previously reported [68].

### 3.3. Pre-Incubation of EPC Cells with Drgal1-L2 Hinders IHNV Adhesion

**Figure 3:**
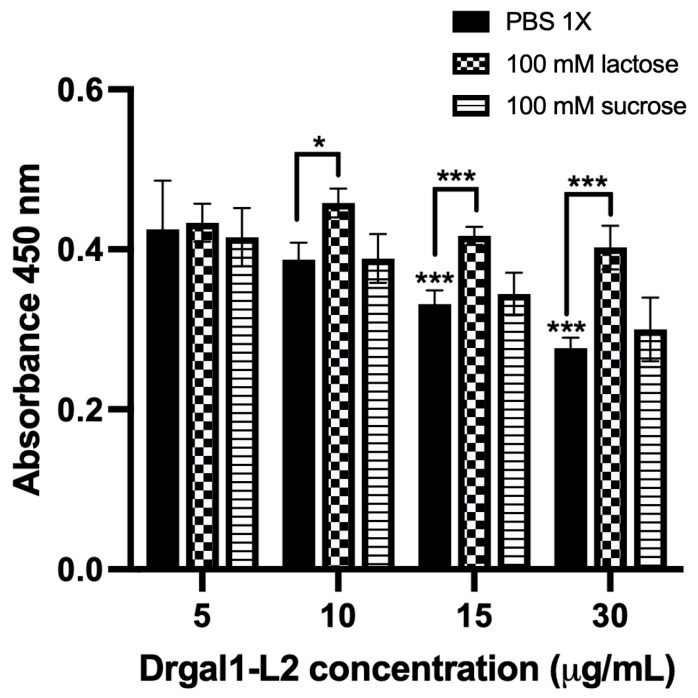
Binding of Drgal1-L2 to the EPC cell surface hinders IHNV adhesion. EPC cells grown in a 96-well microtiter plate (100% confluence), were incubated with various concentrations (5–30 µg/mL) of rDrgal1-L2 only (PBS) or Drgal1-L2 that had been pre-incubated with 100 mM of either lactose (100 mM lactose) or sucrose (100 mM sucrose) for 1 h at room temperature. After washes, the cell monolayer was incubated with IHNV (5 × 10^6^ PFU/mL) in culture medium for 30 min, followed by three washes to remove the unbound virus. Adhesion of IHNV to the EPC cell monolayer was assessed with HRP-conjugated streptavidin (Thermo Scientific), followed by TMB substrate and stopped by addition of 1 M HCl. Average absorbance values read at 450 nm in triplicate for each sample from three independent experiments are shown. Bar graphs show average of data +/− SEM. Significance is shown * *p* < 0.01 and *** *p* < 0.0005.

In a previous study we observed that IHNV adheres to the EPC cell surface, and that pre-incubation of the IHNV virions with Drgal1-L2 would hinder IHNV adhesion [68]. Thus, we now addressed the question of if, reciprocally, the binding of Drgal1-L2 to the cell surface carbohydrate moieties identified in the above-described EPC glycotyping would have similar inhibitory effect on IHNV adhesion to the cell surface. For this, we pre-incubated an EPC monolayer with Drgal1-L2 to investigate if it would also hinder IHNV adhesion, and if so, if it is carbohydrate-specific. The results (Figure 3) showed that pre-incubation of an EPC monolayer with Drgal1-L2 hinders IHNV adhesion in a dose-dependent and carbohydrate-specific manner.

### 3.4. Pre-Incubation of EPC Cells with Drgal1-L2 Limits IHNV Infection

**Figure 4:**
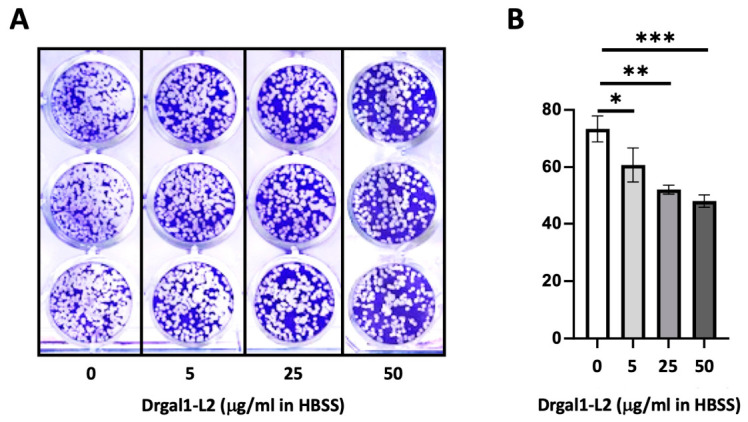
Binding of Drgal1-L2 to the EPC cell surface hinders IHNV infection. (**A**) Confluent monolayers of EPC cells grown in 24-well flat-bottom plates were incubated for 2 h with doubling dilutions of recombinant Drgal1-L2 (5, 25, or 50 mg/mL) or HBSS alone, washed twice with HBSS, infected with IHNV (200 PFU) in HBSS, washed three times with HBSS, and then incubated for 5–7 days under a 0.75% methyl cellulose overlay. Cells were fixed and stained with a 1% crystal violet solution and examined for plaques. Representative data from three independent experiments are shown. (**B**) Plaque areas were quantified by absorbance measurements of dried crystal violet eluted with 33% acetic acid. Bar graphs show average of triplicate data +/− SEM. Significance is shown * *p* < 0.01, ** *p* < 0.005, and *** *p* < 0.0005.

As viral adhesion to the cell surface constitutes the first step in the infection process, we next investigated if in addition to hindering IHNV adhesion, the binding of Drgal1-L2 to the EPC surface would also limit IHNV infection. For this, we carried out plaque formation assays, in which an EPC monolayer was pre-incubated with Drgal1-L2, to examine if the bound galectin would impact the extent of IHNV infection, by comparing the plaque formation in EPC monolayers incubated in the absence of Drgal1-L2. The results (Figure 4A,B) showed that pre-incubation of an EPC monolayer with Drgal1-L2 significantly hindered plaque formation by IHNV infection.

These results brought up the question about the nature of the molecule(s) on the EPC cell surface that IHNV adheres to, and that can also be recognized by Drgal1-L2. Previous work by others found that IHNV adheres to a truncated fibronectin on the fish epithelial cell surface [44]. Given that fibronectin is a glycoprotein also recognized as a strong ligand for galectins, this observation would be consistent with the abovementioned results of hindrance of viral adhesion by Drgal1-L2. However, other cell surface glycoproteins such as well-established galectin ligands CD147 and β1integrin, have also been reported as receptors for viruses [75,76,77,78,79]. Therefore, we considered the possibility that in addition to fibronectin, IHNV also adheres to CD147 and β1integrin. In other words, the hindrance of IHNV adhesion to the EPC cell surface by Drgal1-L2 may also be due at least in part to binding of Drgal1-L2 to cell surface β1integrin and/or CD147. For this, we tested in a solid phase assay the potential binding of IHNV to the three glycoproteins, using BSA as a control.

### 3.5. IHNV Virions Adhere to Immobilized Purified Fibronectin and β1integrin, but Not to CD147

**Figure 5:**
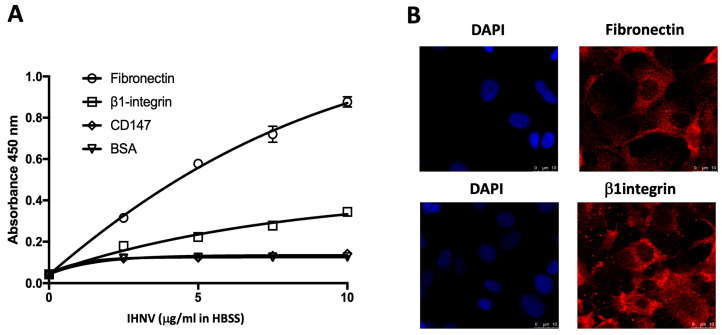
IHNV adheres to selected purified cell surface glycoproteins. Fibronectin and β1integrin are displayed on the EPC cell surface: (**A**) Each well of a 96-well microtiter plate was coated with 100 µL of 5 µg/mL of either fibronectin (circles), β1integrin (squares), CD147 (diamonds), or BSA (triangles), and then incubated with increasing concentrations of purified biotinylated IHNV (0–10 µg/mL). Virus adhesion was detected with HRP conjugated streptavidin (Pierce) followed by TMB substrate and stopped by addition of 1 M HCl. Absorbance values were read at 450 nm. Average of triplicate data (+/−) standard error of the mean standard deviation is represented. (**B**) EPC cells display cell surface glycoproteins strongly cross-reactive with fibronectin and β1integrin. EPC cells (60–70% confluency) on 8-well chamber slides were fixed and incubated with anti-fibronectin (top right) or anti-β1integrin (bottom right) followed by Alexa 555 (anti-rabbit) and counterstained with DAPI (left panels). Slides were imaged using an Echo Revolve Microscope. Representative images from three independent experiments are shown.

We carried out experiments to investigate the potential binding of IHNV to immobilized glycoproteins: commercial purified bovine fibronectin and recombinant β1integrin and CD147, which are well-established galectin ligands that could constitute the basis for the Drgal1-L2-mediated hindrance of IHNV adhesion. The results (Figure 5A) showed that IHNV adheres to immobilized fibronectin and less effectively to β1integrin but does not adhere to CD147.

### 3.6. Fibronectin and β1integrin Are Present and Exposed on the Surface of the EPC Cells

Next, we examined if fibronectin and β1integrin, two candidate receptor glycoproteins for IHNV, are present and displayed on the surface of EPC cells for viral adhesion. For this, we used anti-fibronectin and anti-β1integrin specific antibodies to carry out immunofluorescence analysis on intact EPC cells. Results revealed that both glycoproteins are exposed on the EPC cell surface (Figure 5B).

### 3.7. The Recombinant β1integrin Expressed in CHO Cells Is Glycosylated and Displays LacNAc Moieties

To rationalize the basis for the Drgal1-L2-mediated hindrance of IHNV adhesion and infection of EPC cells we needed to confirm that Drgal1-L2 binds to either or both glycoproteins fibronectin and β1integrin. While the fibronectin we used for these experiments is the authentic bovine fibronectin, and thus naturally glycosylated, the β1integrin is a recombinant glycoprotein expressed in CHO cells. Therefore, we performed glycotyping with plant lectins of well-established specificity to confirm that it is equally glycosylated as the authentic natural equivalent and displays LacNAc moieties that could be recognized and bound by galectins. The results confirmed that the recombinant β1integrin expressed in CHO cells is correctly glycosylated, and thus, as revealed by the strong binding of ECA, both fibronectin and β1integrin display the disaccharide LacNAc, the preferred ligand for proto-type galectins (Table 1).

### 3.8. Drgal1-L2 Binds to Immobilized Purified Fibronectin and β1integrin

**Figure 6:**
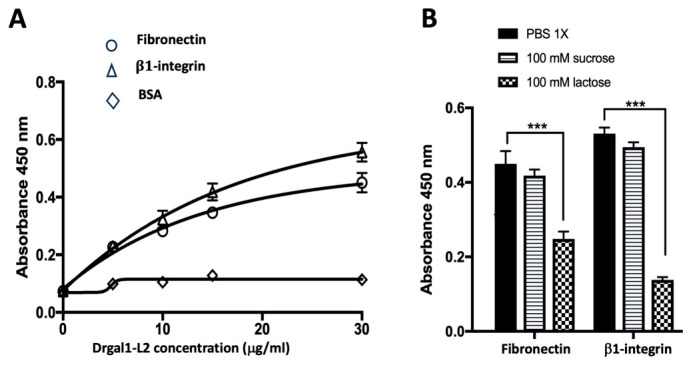
Drgal1-L2 binds specifically to purified bovine fibronectin and recombinant β1integrin expressed in CHO cells: (**A**). Fibronectin, β1integrin and BSA were immobilized on 96-well plates and incubated with increasing concentrations of Drgal1-L2 (0–30 µg/mL) to detect the Drgal1-L2 binding in a solid phase assay. Average absorbance values at 450 nm of triplicate data (+/−) standard error of the mean are shown. (**B**). The binding of Drgal1-L2 (30 µg/mL) to immobilized purified cell surface glycoproteins (fibronectin and β1integrin) in the presence of either 100 mM of lactose, 100 mM of sucrose, or buffer only (PBS 1X) was assayed as above. Average absorbance values at 450 nm of triplicate data (+/−) standard error of the mean are shown. Significance is shown *** *p* < 0.0005.

Next, we carried out a solid-phase assay to examine if Drgal1-L2 binds to the purified bovine fibronectin and recombinant glycoprotein β1integrin. For this, the glycoproteins were immobilized and tested for binding by increasing concentrations of Drgal1-L2, using lactose and sucrose as positive and negative control inhibitors, respectively. The results confirmed that Drgal1-L2 binds to both bovine fibronectin and recombinant β1integrin in a dose-dependent (Figure 6A) and carbohydrate-specific manner (Figure 6B).

### 3.9. SPR Analysis of Drgal1-L2 Binding to the Two Candidate IHNV Receptor Glycoproteins, Fibronectin and β1integrin

**Figure 7:**
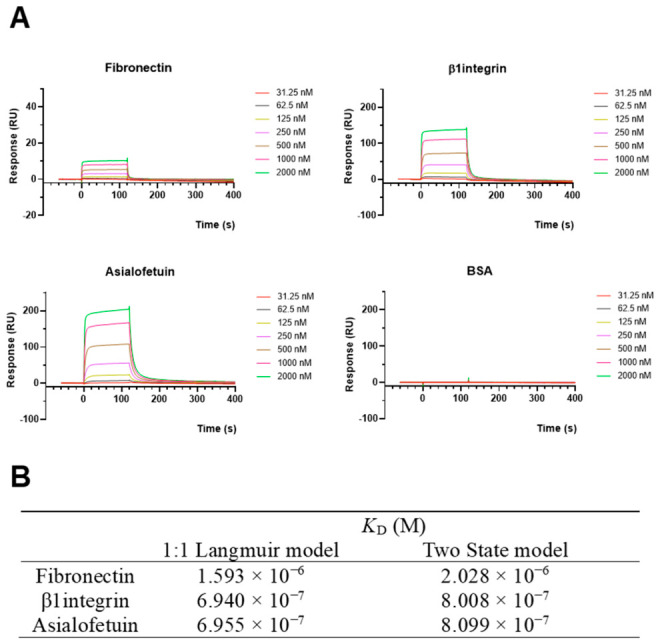
SPR analysis of Drgal1-L2 binding to purified glycoproteins. The zebrafish galectin isoform Drgal1-L2 binds to fibronectin and β1integrin in a reversible manner: (**A**) SPR analysis of Drgal1-L2 binding to fibronectin and β1integrin, compared to the positive and negative controls, asialofetuin and BSA, respectively. Glycoproteins were immobilized on a CM5 sensor chip and Drgal1 was run as analytes at 2× serial dilutions starting from 2000 nM to 31.25 nM. The sensorgram fitted using the 1:1 Langmuir model is shown. (**B**) Equilibrium dissociation constants (*K*_D_) from the experimental data fitted using two different models (1:1 Langmuir model and two-state model) with the Biacore T200 Evaluation Software (GE Healthcare).

Subsequently, we assessed the relative strength of the binding of Drgal1-L2 to the two candidate IHNV receptor glycoproteins, fibronectin and β1integrin, using ASF and BSA as controls, by surface plasmon resonance (SPR) analysis. To obtain the equilibrium dissociation constant (*K*_D_), the experimental data were fitted using two different models with the Biacore T200 Evaluation Software (GE Healthcare): the 1:1 Langmuir model (Figure 7A) and the two-state model (Supplementary Figure S1). The SPR results strongly suggested that Drgal1-L2 binds to the two candidate IHNV receptor glycoproteins, fibronectin and β1integrin, in a carbohydrate-specific manner, as it also binds strongly to the control ASF but not to BSA. The *K*_D_ values obtained from the two models (Figure 7B) were comparable and showed consistent trends. Both sensorgrams fitted quality metrics (Rmax values and χ^2^ values) as low χ^2^ values indicated good agreement between the experimental data and the fitted models. The estimated *K*_D_ values fall within the micromolar range, which is typical for many extracellular protein–ligand interactions [13]. Considering the estimated concentrations of Drgal1-L2 and its ligands in mucus or at epithelial surfaces, these affinities are compatible with physiologically relevant binding.

### 3.10. Binding of Drgal1-L2 to Either Immobilized Fibronectin or IHNV Virions Hinders Adhesion of IHNV Virions to Fibronectin in a Carbohydrate-Dependent and Specific Manner

**Figure 8:**
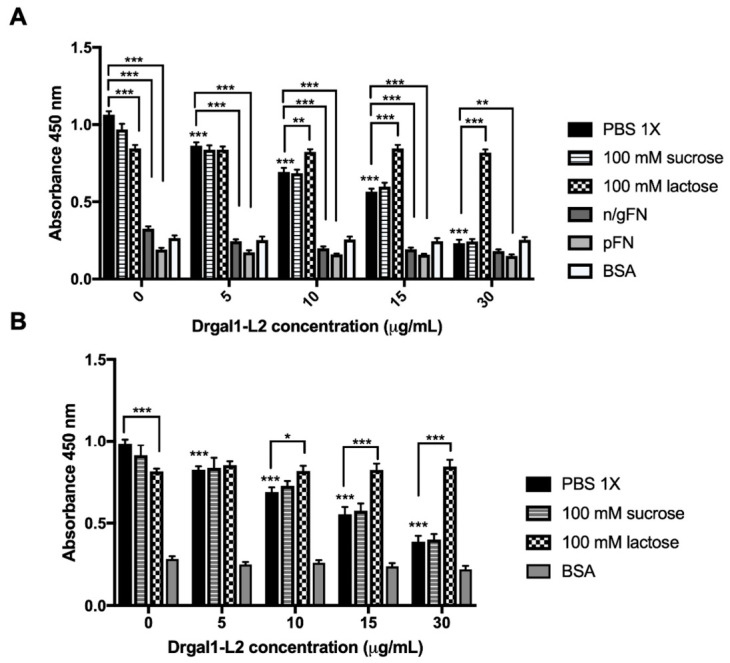
Binding of Drgal1-L2 to either immobilized fibronectin or IHNV hinders IHNV adhesion to fibronectin in a dose–response and carbohydrate-dependent manner. (**A**) 5 µg/mL of FN, nFN, n/gFN and pFN were immobilized and pre-incubated with increasing concentrations of Drgal1-L2 (0–30 µg/mL) in the presence of either PBS, 100 mM lactose or 100 mM sucrose, followed by addition of biotinylated IHNV (7.5 µg/mL) for incubation. Virus binding was detected with HRP-conjugated streptavidin at (0.15 mg/mL concentration). Average absorbance values at 450 nm of triplicate data (+/−) standard error of the mean is represented in graph. * *p* < 0.05, ** *p* < 0.005, *** *p* < 0.0005 for lactose vs. PBS, Control (0 µg/mL) vs. increasing Drgal1-L2 concentrations, FN vs. n/g FN, and FN vs. pFN. (**B**) FN (5 µg/mL) immobilized on a 96-well microtiter plate were incubated with biotinylated IHNV (7.5 µg/mL) which was pre-incubated with increasing concentrations of Drgal1-L2 (0–30 µg/mL) in the presence of either PBS, 100 mM lactose or 100 mM sucrose. Virus binding was detected as above A, and average absorbance values at 450 nm of triplicate data (+/−) standard error of the mean are shown. Adhesion of biotinylated IHNV onto immobilized BSA (5 µg/mL) was included as negative control.

Having confirmed that fibronectin can function as a cell surface receptor for IHNV, and that it is a ligand for Drgal1-L2, next we investigated if the binding of Drgal1-L2 to either fibronectin or IHNV inhibits IHNV adhesion to fibronectin, in a carbohydrate-dependent and specific manner. For this, we carried out solid phase assay experiments to examine if Drgal1-L2 binding to the purified bovine fibronectin, either untreated or treated with glycohydrolases, or to IHNV virions hinders IHNV adhesion to immobilized fibronectin. The results (Figure 8) showed that binding of Drgal1-L2 to the immobilized fibronectin (Figure 8A) or to the IHNV virions (Figure 8B) hinders the adhesion of the IHNV virions to the immobilized fibronectin. For either case, the lactose controls prevented the galectin-mediate hindrance of IHNV adhesion to fibronectin, whereas the sucrose control had no effect.

### 3.11. Binding of Drgal1-L2 to Either Immobilized Recombinant β1integrin or IHNV Virions Hinders Adhesion of IHNV Virions to β1integrin in a Carbohydrate-Dependent and Specific Manner

**Figure 9:**
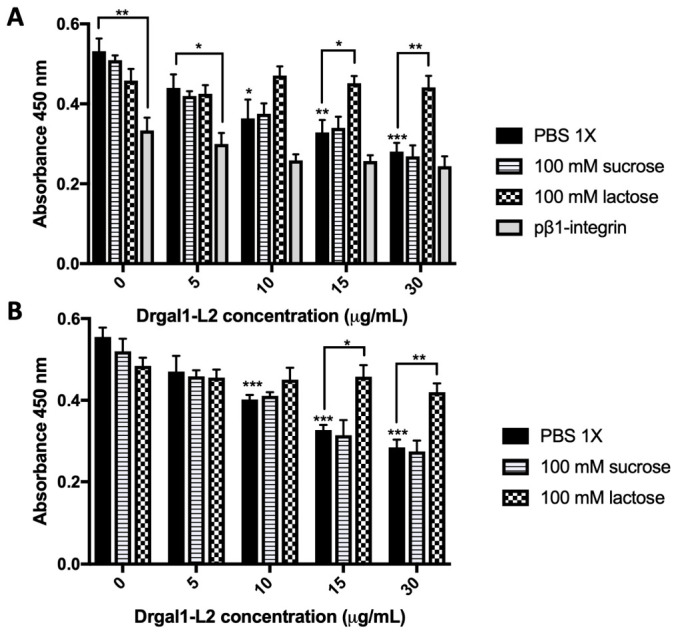
Binding of Drgal1-L2 to either immobilized β1integrin or IHNV hinders IHNV adhesion to β1integrin in a dose–response and carbohydrate-dependent manner. (**A**) 2.5 µg/mL of immobilized β1integrin or deglycosylated β1integrin (pβ1integrin) were incubated with increasing concentrations of Drgal1-L2 (0–30 µg/mL) in the presence of either PBS, 100 mM lactose or 100 mM sucrose, followed by addition of biotinylated IHNV (15 µg/mL) for incubation. Virus binding was detected with HRP-conjugated streptavidin at (0.15 µg/mL concentration). Average of triplicate data (+/−) standard error of the mean is represented in graph. * *p* < 0.05, ** *p* < 0.005, *** *p* < 0.0005 for lactose vs. PBS 1X, Control (0 µg/mL) vs. increasing Drgal1-L2 concentrations, and β1integrin vs. pβ1integrin. (**B**) ELISA was performed to detect the binding of biotinylated IHNV (15 µg/mL) pre-incubated with increasing concentrations of Drgal1-L2 (0–30 µg/mL) to 2.5µ/mL of β1integrin immobilized on a 96-well microtiter plate. IHNV was incubated with Drgal1-L2 in the presence of either PBS, 100 mM lactose or 100 mM sucrose before being added to the plate.

Next, having found that β1integrin is also a ligand for Drgal1-L2 we carried out similar experiments to investigate if Drgal1-L2 specific binding to immobilized recombinant β1integrin (either untreated or glycohydrolase(p)-treated as specificity controls) or IHNV virions inhibits IHNV adhesion to β1integrin. The results showed that either Drgal1-L2 binding to the β1integrin (Figure 9A) or to the IHNV virions (Figure 9B) hinders the adhesion of the IHNV virions to β1integrin. For either interaction of Drgal1-L2 with β1integrin or IHNV, the lactose controls prevented the galectin-mediate hindrance of IHNV adhesion to β1integrin, whereas the sucrose control had no effect.

### 3.12. Anti-Fibronectin and Anti-β1integrin Antibodies Co-Localize with Drgal1-L2 on the EPC Cell Surface, and Hinder IHNV Adhesion to EPC Cells

**Figure 10:**
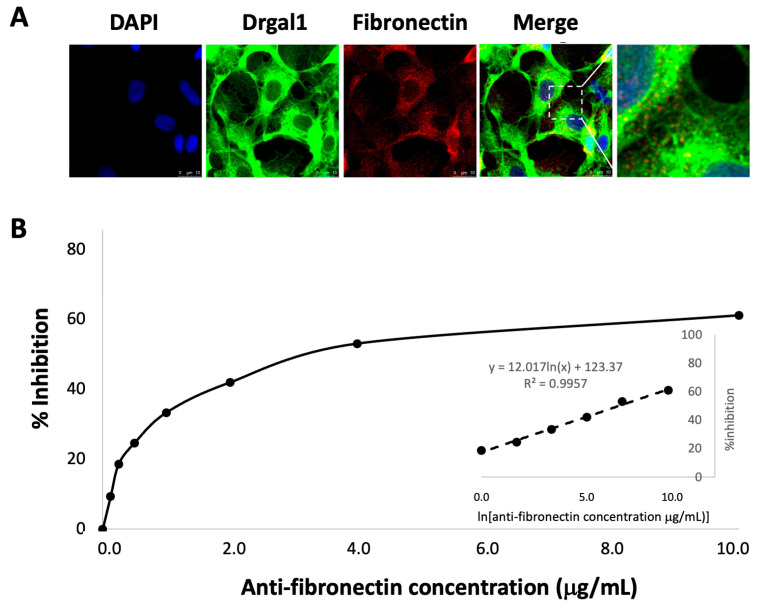
Anti-fibronectin antibodies co-localize with Drgal1-L2 bound to the EPC surface, and hinders IHNV adhesion to an EPC monolayer. (**A**) Co-localization of anti-fibronectin antibodies with Drgal1-L2 on the EPC surface: EPC cells (60–70% confluency) grown on 8-well chamber slides were fixed and blocked, then incubated with Drgal1L2 (100 µg/mL) for 6 h, followed by immunostaining with anti-fibronectin antibodies (1:500) and incubated for 1 h, followed by the appropriate secondary antibodies. The nuclei were counter-stained with DAPI (1:2000) for 2 min and the images were captured with confocal microscopy (Leica Microsystems) at 63X magnification. Areas of colocalization of bound Drgal1-L2 (green) and anti-fibronectin (red) appeared yellow in “Merge”, and are shown at higher magnification in the insets. Images are representative of multiple fields of view from at least three independent experiments. (**B**) Binding of anti-fibronectin antibodies to an EPC monolayer partially hinders IHNV adhesion to the EPC cell surface: EPC cells (100% confluence) grown in 96-well microtiter plates were incubated with increasing concentrations (0–10 µg/mL) of anti-fibronectin antibodies followed by IHNV (5 × 10^6^ PFU/mL) in culture medium or culture medium only (negative control) for 30 min. Adhesion of IHNV to the EPC cell monolayer was assessed with HRP-conjugated streptavidin with TMB substrate and the reaction was stopped by adding 1 M HCl. Absorbance values were read at 450 nm. The percentage of inhibition was calculated over the adhesion without antibody (culture medium only) and plotted. The anti-fibronectin antibodies bound to the EPC monolayer surface hinder adhesion of IHNV virions in 60–62% at the highest concentration.

**Figure 11:**
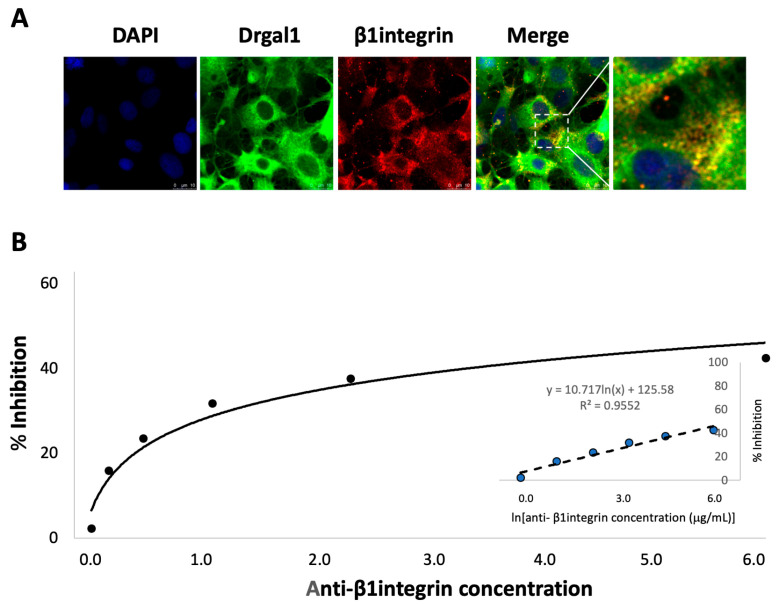
Anti-β1integrin antibodies co-localize with Drgal1-L2 bound to the EPC surface, and hinders IHNV adhesion to an EPC monolayer. (**A**) Co-localization of anti-β1integrin antibodies with Drgal1-L2 on the EPC surface: EPC cells (60–70% confluency) on 8-well chamber slides were fixed and blocked, then incubated with Drgal1-L2 (100 µg/mL) for 6 h, followed by immunostaining with anti-β1integrin antibodies and appropriate secondary antibodies. The nuclei were counter-stained with DAPI. Images were captured with confocal microscopy (Leica Microsystems) at 63X magnification. Areas of colocalization of bound Drgal1-L2 (green) and anti-β1integrin (red) appear yellow in the insets. Images are representative of multiple fields of view from at least three independent experiments. (**B**) Binding of anti-β1integrin antibodies to an EPC monolayer partially hinders IHNV adhesion to the EPC cell surface: EPC cells (100% confluence) grown in 96-well microtiter plates were incubated with increasing concentrations (0–6 µg/mL) of anti-β1integrin antibodies, followed by IHNV (5 × 10^6^ PFU/mL) in culture medium or culture medium only (negative control) for 30 min. Adhesion of IHNV to the EPC cell monolayer was assessed with HRP-conjugated streptavidin, using TMB substrate and the reaction was stopped by adding 1 M HCl. Absorbance values were read at 450 nm. The percentage of inhibition was calculated over the adhesion without antibody (culture medium only) and plotted. The anti-β1integrin antibodies bound to the EPC monolayer surface hinder adhesion of IHNV virions in a relatively lower % as compared to anti-fibronectin antibodies (41–42% at the highest concentration).

Once we had demonstrated the capacity of Drgal1-L2 to hinder IHNV adhesion to immobilized purified glycoproteins that may function as potential glycoprotein IHNV receptors at the cell surface, we carried out similar in vitro testing of Drgal1-L2 activity using EPC monolayers instead of immobilized glycoproteins to examine if Drgal1-L2 binding to the cell surface receptor(s) can hinder IHNV attachment to the EPC cell surface. However, the glycotyping experiments showed that EPC cells display a complex glycocalyx with exposed LacNAc, suggesting that there is a variety of glycoproteins that could function as IHNV receptors. Thus, to ensure that in the viral adhesion-inhibition experiments we were specifically targeting either the cell surface fibronectin or β1integrin, we first carried out co-localization experiments of Drgal1-L2 with anti-fibronectin- or anti-β1integrin-specific antibodies by immunofluorescence on EPC cell monolayers. Second, to investigate the possibility that fibronectin or β1integrin can act as receptors for IHNV on the EPC cell surface we used specific anti-fibronectin or anti-β1integrin antibodies to pre-treat the EPC and test if they hinder IHNV binding. To verify that the binding of anti-fibronectin or anti-β1integrin could hinder IHNV adhesion to the target glycoproteins on the cell surface, we first tested their blocking capacity by using purified fibronectin and β1integrin immobilized on 96-well microtiter plates incubated with increasing concentrations of the specific antibody and then assessed the adhesion of biotinylated IHNV. Results showed that pre-incubation of purified fibronectin or β1integrin with the specific antibody hinders IHNV adhesion in a dose-response manner, by approximately 80% for fibronectin (Supplementary Figure S2) and by 45% for β1integrin (Supplementary Figure S3) at the highest antibody concentration tested. Results of the co-localization experiments revealed that the anti-fibronectin antibody co-localized with Drgal1-L2 on EPC cells (Figure 10A), and that it inhibits IHNV adhesion to EPC up to about 60% at the highest concentration tested (Figure 10B). Similarly, the anti-β1integrin antibody co-localized with Drgal1-L2 on the EPC cell surface (Figure 11A) and inhibited IHNV adhesion to EPC up to about 40% at the highest concentration tested (Figure 11B). The latter results added support to the proposal that in addition to fibronectin, β1integrin may also function as a cell surface receptor for IHNV virions.

### 3.13. Binding of Drgal1-L2 to Either IHNV or Immobilized Zebrafish Epidermal Mucus Hinders Viral Adhesion to Mucus in a Dose–Response and Carbohydrate Dependent Manner

**Figure 12:**
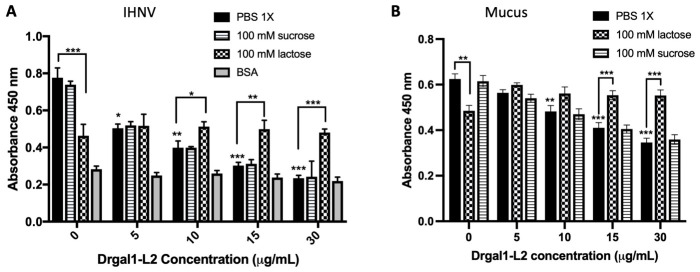
Binding of Drgal1-L2 to either IHNV or immobilized zebrafish epidermal mucus hinders viral adhesion to mucus in a dose–response and carbohydrate-dependent manner: (**A**) The potential role of Drgal1-L2 binding to IHNV in preventing viral adhesion to zebrafish epidermal mucus was investigated by ELISA: Mucus (100 µg/mL) was immobilized on 96-well microtiter plates. Biotinylated IHNV virions pre-incubated with increasing concentrations of Drgal1-L2 (0–30 µg/mL) in either PBS, 100 mM sucrose, or 100 mM lactose for 1 h were added to the wells with immobilized zebrafish mucus. IHNV adhesion to mucus was detected with HRP-conjugated streptavidin at (0.15 mg/mL concentration). Adhesion of biotinylated IHNV onto immobilized BSA (5 µg/mL) was included as negative control. (**B**) The potential role of Drgal1-L2 binding to zebrafish epidermal mucus in preventing IHNV adhesion to the immobilized mucus was investigated by ELISA: Mucus was immobilized in 96-well microtiter plates as described in A above, and incubated with increasing concentrations of Drgal1-L2 (0–30 µg/mL) in either PBS 1X, 100 mM sucrose, or 100 mM lactose, before biotinylated IHNV in PBS was added to the wells with immobilized zebrafish mucus. IHNV adhesion to mucus was detected as described in A above. Adhesion of biotinylated IHNV onto immobilized BSA (5 µg/mL) was included as negative control. In both experiments, average of triplicate data (+/−) standard error of the mean standard deviation is represented: * *p* < 0.05, ** *p* < 0.005, *** *p* < 0.0005 for lactose vs. PBS 1X, and control (0 µg/mL) vs. increasing Drgal1-L2 concentrations.

Lastly we investigated the possibility that Drgal1-L2 present in the epidermal mucus could function as a defense factor by hindering adhesion of IHNV to the epidermal mucus glycans and therefore prevent the virions from accessing to the cell surface. For this we first preincubated IHNV with increasing concentrations of Drgal1-L2 and tested its adhesion to immobilized zebrafish epidermal mucus (Figure 12A). Second, we preincubated the immobilized zebrafish epidermal mucus with increasing concentrations of Drgal1-L2 and tested adhesion of IHNV (Figure 12B). Results showed that treating either IHNV or the immobilized mucus significantly reduced the adhesion of the virions to mucus in a dose–response and carbohydrate-dependent manner (Figure 12A,B). This observation suggests that by binding to the virion envelope glycans or mucus mucins, Drgal1-L2 present in the epidermal mucus film can further prevent the access of IHNV virions to the underlying epithelial cell surface.

## 4. Discussion

The initial stage for viral infection of mucosal epithelia consists of adhesion of the virus to receptors on the surface of epithelial cells from tissues located at the interface of the host with the environment that can be exposed to the infective virions [80,81]. In turn, the host employs multiple defense strategies to prevent access of the virions to the cell surface receptors [82,83,84]. Among the innate immune factors that the host has evolved to prevent, neutralize, or clear viral infections, galectins have been recently identified not only as recognition molecules but also as effector factors [28,29,30,31,32,85]. Galectins can bind to glycans on the surface of potential pathogens, such as viruses, bacteria, and eukaryotic parasites to prevent their adhesion and entry to the host [28,29,30,31,32,33]. If an infection has been established, galectins can agglutinate, immobilize, opsonize, and in some cases directly neutralize or kill the potential pathogen, and mediate their clearance from the internal milieu [28,29,30,31,32,33].

In this study we investigated the possibility that the binding of extracellular Drgal1-L2 to glycoproteins on surface of fish epithelial cells that may function as receptors for IHNV may contribute to block adhesion of IHNV virions to the surface of fish epithelial cells. This question arose from our previous study in which we observed that Drgal1-L2 was expressed and secreted by fish epithelial cells, and could bind to the IHNV virion glycosylated envelope in a dose-dependent and carbohydrate-specific manner [68]. Incubation of IHNV virions with Drgal1-L2 inhibited in about 40–50% viral attachment to the fish epithelial cell membrane [68]. The resolution of the structure of Drgal1-L2 and computer modeling enabled us to propose a mechanism by that Drgal1-L2 could disrupt the viral spike fusion with the host cell membrane [69]. We also observed, however, that Drgal1-L2 could also bind to the cell membrane of fish epithelial cells [68]. Thus, we hypothesized that in addition to the binding to the IHNV envelope and hindering virion-receptor interactions as we previously reported [68], the binding of the secreted/extracellular Drgal1-L2 to glycans that function as viral receptors on the epithelial cell surface would constitute either an alternative or synergistic Drgal1-L2-mediated mechanism to block access of the IHNV virions to the epithelial cell surface for adhesion, entry, and infection. Given that cell surface fibronectin has been identified as the receptor for IHNV in fish epithelial cells, we initially focused our efforts on investigating the potential effects of Drgal1-L2 binding to fibronectin in IHNV adhesion. As the epithelial cell surface displays other glycoproteins that could function as alternative viral receptors or co-receptors, however, we examined the possibility that the binding of Drgal1-L2 to additional cell surface glycoproteins reported as receptors for rhabdovirus such as β1integrin and CD147, could also interfere with IHNV adhesion/infection.

In vertebrates, including fish, mucosal surfaces such as the respiratory, oral, and gut epithelia are coated with a continuous film of mucus that functions both as a mechanical and bioactive defense barrier against microbial or viral infection [47,48,49,50,51,52,53,54,86,87,88]. In fish, in addition to the gut and olfactory mucosal surfaces, the epithelia of gills and skin are equally coated with a mucus film [89,90,91]. Viruses often adhere to the mucus film coating mucosal surfaces [92], and although this interaction is a critical aspect of the protective function of the epidermal mucus, some viruses have evolved to bypass these defenses [93]. Many viruses, including influenza, SARS-CoV-2, and HPV, bind to mucin glycoproteins within the mucus barrier, often as a first step in the infection process, and afterwards via enzymatic action of enzymes they make their way to the cell surface receptors [94]. Therefore, given that the mucosal epithelia of zebrafish are coated with a film of epidermal mucus [95], we also explored the possibility that binding of Drgal1-L2 present in the epidermal mucus to mucus glycans such as soluble mucins could also contribute to hindering access of the IHNV virions to the epithelial cell surface.

To test our hypotheses, we used an in vitro model system consisting of an epithelial fish cell line and epidermal mucus collected from adult zebrafish. This experimental format would enable us to conduct the pertinent experiments under controlled conditions, to be followed by in vivo gene silencing/editing studies currently ongoing in our laboratory. The Drgal1 isoform expression profiles of the EPC and ZFL cell lines closely reflected their expression patterns of zebrafish mucosal tissues such as skin and gills that we recently reported [96] and enabled the selection of the Drgal1-L2 isoform for all further experiments. The characterization of the glycocalyx by lectin glycotyping further confirmed the EPC cell line as a suitable model system, since it was virtually identical to the ZFL cell line, and displayed cell surface galactose, GalNAc, sialic acid, and mannose at the cell surface. The presence of galactose suggested that these were the sugar moieties recognized by Drgal1-L2 on the cell surface. Galactosylated glycans on the cell surface are mostly glycoproteins and glycolipids that display terminal non-reducing LacNAc, as well as internal LacNAc units, a disaccharide which is the preferred ligand for the mammalian proto type galectins [13]. Similar binding preference has also been well established for proto type galectins from bony fish such as Msgal1-L1 from the striped bass *Morone saxatilis* [97] and the zebrafish Drgal1-L2 as we recently reported [96], the tongue sole *Cynoglossus semilaevis* [98], and the yellow croaker *Larimichthys crocea* [99]. The non-reducing terminal galactose moiety of LacNAc is frequently sialylated in either 2-3 or 2-6 linkage [100]. Proto type galectins like galectin1 can bind equally well to the non-sialylated LacNAc and the sialylated disaccharide in α2-3, but sialic acid linked in α2-6 blocks galectin binding [101].

The hindrance of IHNV adhesion by pre-incubation of the EPC monolayer with Drgal1-L2 provided support for our hypothesis that the binding of Drgal1-L2 present in epidermal mucus to cell surface glycans that can function as viral receptors may contribute as an alternative or additive protective mechanism against viral adhesion to the host epithelial cell surface. The defensive roles of galectins against viral infection have been reported to be mediated by various mechanisms, including the binding of galectins to the viral glycosylated envelope, such as of influenza A and Dengue viruses [28,30,32,102,103,104,105,106]. It should be kept in mind that several studies have shown that in contrast to the above, however, the binding of galectins to the envelope of certain viruses such as Epstein–Barr virus (EBV), human immunodeficiency type 1 (HIV-1), Nipah, enterovirus 71 (EV71), human T cell leukemia type 1 (HTLV-1), and herpes simplex (HSV-1) can facilitate viral adhesion and infection by various mechanisms, including crosslinking the virus to their glycosylated receptors at the cell surface [107,108,109,110,111,112,113,114,115,116,117,118,119,120,121,122,123].

The observation in this study that Drgal1-L2 binding to the IHNV virion glycosylated envelope hinders viral adhesion to epithelial cell monolayers, confirmed that the binding of extracellular Drgal1-L2 to the IHNV glycosylated envelope can function as a preventive mechanism against IHNV host cell adhesion, and potentially, infection. These results are consistent with prior observations by our lab and others that the binding of Gal1 or Gal3 to viruses like influenza A and Dengue can inhibit or at least ameliorate infection [68,69,102,103].

The hindering of viral adhesion by galectin binding to viral receptors, however, has not been addressed in comparable detail. Thus, in this study we aimed at exploring the capacity of Drgal1-L2 for preventing IHNV infection by binding to its cognate cell surface receptor(s) to potentially identify (unravel) a novel defense mechanism against viral infection of mucosal epithelia. The host cellular receptor for IHNV and other rhabdoviruses such as viral hemorrhagic septicemia virus (VHSV) in epithelial cells has been identified as fibronectin [44,124], and our observation that purified IHNV virions can adhere to purified immobilized fibronectin confirmed these reports. Importantly, our observation that Drgal1-L2 also binds to immobilized purified fibronectin enabled us to rationalize the hindrance of IHNV adhesion to purified fibronectin by prior exposure of the immobilized glycoprotein to Drgal1-L2. Similarly, the hindrance of IHNV adhesion to the cell surface by pre-exposure of the EPC cell monolayer to either Drgal1-L2 or a specific anti-fibronectin antibody added support to the proposed basis for these interactions. The latter results should be interpreted with caution, however, as the antibody-mediated hindrance of IHNV adhesion could also be due to steric hindrance or non-specific interference with surface accessibility. Fibronectin is a glycoprotein present on the cell surface, ECM, and plasma involved in basic functions in cell adhesion, migration, growth and differentiation that are essential to early embryonic development, angiogenesis, and wound repair. It is no surprise then that it has been highly conserved along evolution of invertebrate and vertebrate lineages, from the earliest multicellular organisms (parazoa, e.g., sponges) to man [125,126,127,128,129,130,131,132]. The typical mammalian fibronectin structure consisting of the typical arrangements of domains formed by type I-III repeats, however, appears in the agnathans (jawless chordates such as lampreys and hagfish) and is expressed in all vertebrate taxa, with structural variability mostly limited to alternative splicing [125,126,127,128]. The zebrafish expresses a unique truncated fibronectin which is homologous to the mouse and mammalian equivalents, and shares over 50% of sequence identity [128] (Supplementary Figure S4). Fibronectin is highly N-glycosylated and displays LacNAc moieties [133] mostly as poly-N-acetyllactosamine chains, which are key to its multiple extracellular functions in cell adhesion and migration [134]. Given that LacNAc moieties are also the preferred ligands for proto-type galectins including Drgal1-L2, the binding of galectins to fibronectins in various functional contexts has been widely reported [135]. Fibronectin has been identified not only as the archetype receptor for bacterial adhesins [136,137,138] but can also function as a receptor for several viruses by direct interactions with the viral capsule or envelope proteins or by facilitating virus entry by indirect mechanisms. For example, influenza A virus, hepatitis B virus, HIV-1, Abelson murine leukemia virus, and enterovirus A71 use fibronectin directly or indirectly to adhere and/or enter the host cell [139,140,141,142,143,144]. Regarding rhabdoviruses, those infecting fish such as IHNV and VHSV use cell surface fibronectin as a receptor [44,124], whereas mammalian rhabdoviruses such as rabies and vesicular stomatitis virus (VSV) use different adhesion receptors and entry mechanisms [145].

In addition to fibronectin, other cell surface N- and O-linked glycans also display LacNAc moieties to which galectins strongly bind such as CD147, β1integrin, VEGEFR-1/-2, CD44, CD45 and others that have been reported as strong ligands for galectins have also been reported as receptors for viruses [76,85,101,146,147,148]. Among these, for example, the rabies virus is a rhabdovirus that uses β1integrin as a receptor [149], whereas hepatitis B and C viruses use CD147 [150]. From these possible candidates we selected β1integrins as they are glycoproteins reported not only as strong ligands for proto-type galectins, but also as receptors or co-receptors for viruses or cell surface components that indirectly facilitate their binding and/or entry [151,152,153,154,155] and particularly the rabies virus [149]. We also tested CD147 as it has been reported to function as a cell surface receptor for several viruses, including measles [76], and chikungunya [146], and more recently SARS-CoV-2 [156], although this is still a matter of debate [157]. Interestingly, although Drgal1-L2 bound to both immobilized purified glycoproteins, our results showed that IHNV selectively adhered to immobilized purified β1integrin but not to CD147. Like we observed with fibronectin, the selective adherence of IHNV to purified β1integrin was consistent with its potential role as a receptor for IHNV at the cell surface on EPC cells. The inhibition of IHNV adhesion in a dose–response manner by pre-treatment of the EPC cell monolayers with increasing concentrations of anti-β1integrin specific antibodies added support to this possibility. As cautioned above for the interpretation of results obtained with anti-fibronectin antibodies, it should be kept in mind that the anti-β1integrin-mediated hindrance of IHNV adhesion to the EPC surface could also be non-specific. β1integrin is a transmembrane protein that interacts with several ECM components like fibronectin and laminin and has critical roles in signaling and cell differentiation, adhesion, proliferation and migration, in essential functions such as early development and wound healing, as well as tumor angiogenesis and progression [158,159,160]. Like fibronectin, and due to its key roles in cell adhesion, β1integrin is highly conserved in evolution both structurally and functionally, from sponges to mammals, including man [161,162,163,164] (Supplementary Figure S5). The extracellular domains of β1integrin are N-glycosylated at several sites of the polypeptide chain that are critical for proper folding and dimerization but also for interactions with partner proteins such as fibronectin and laminin [165,166,167,168]. The terminal moieties of the β1integrin N-linked oligosaccharides consist mostly of LacNAc as polylactosamines and therefore are recognized by most galectins [166].

The in vitro adherence of IHNV to both purified fibronectin and β1integrin was interesting from the standpoint that some viruses can bind to multiple distinct cell surface receptors [169]. In some cases, the virus uses first a receptor to adhere to the cell surface and this is followed by the interaction with a second receptor or co-receptor that mediates entry into the cell. Such are the cases of herpes simplex virus (HSV) that first binds to cell surface heparan sulfate proteoglycans followed by nectin-1/nectin-2 and the HSV entry mediator [170], and HIV-1 that first adheres to CD4, and subsequently a CCR5 co-receptor that facilitates entry into the host cell [171]. For other viruses, the ability to bind multiple receptors gives them the capacity to infect different cell types and tissues [172,173]. For example, HSV can use HVEM, Nectin-1, or 3-O-sulfated heparan sulfate, as receptors in different cell types [174,175]. Our results suggest that IHNV virions, however, may adhere to both fibronectin and β1integrin on the same epithelial EPC cell. Some viruses can bind to one cell surface protein as a primary receptor to become tethered/attached to the cell surface and a second one as a co-receptor that mediates entry of the virus into the cell [176,177,178]. For example, the Coxsackie virus first adheres to CAR, the specific Coxsackie virus receptor, and enters the cell facilitated by integrins (αvβ3 or αvβ5) [179,180]. Thus, it is possible that fibronectin acts as the primary receptor that tethers and concentrates the IHNV virions on the cell surface, and β1integrin mediates cell entry. Since fibronectin and β1integrin interact with each other at the cell surface [181,182], however, the binding of IHNV to both purified glycoproteins most likely reflects a complex mechanism of viral adhesion and entry into the fish epithelial cell that warrants further investigation. Moreover, the lack of direct binding of IHNV to purified CD147, however, should not rule out its participation as a co-receptor on the adhesion and entry mechanism of IHNV into the epithelial cell. CD147 interacts with both fibronectin and β1integrin, and it is possible that the direct binding of IHNV to either or both glycoproteins is followed by interactions with CD147 that may also modulate the virus adhesion and host cell entry mechanisms [183,184,185,186].

**Figure 13:**
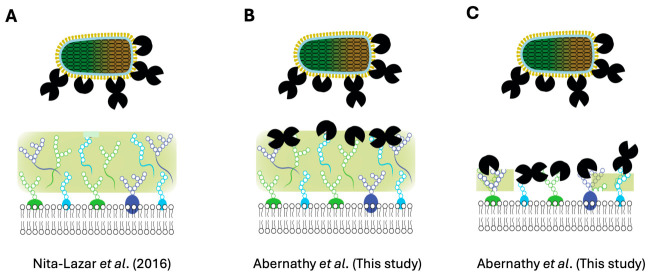
Hypothetical model for the Drgal1-L2-mediated hindrance of IHNV adhesion to fish epithelial cells and epidermal mucus. (**A**) In our previous study [68] we showed that pre-incubating IHNV virions with the zebrafish galectin Drgal1-L2 could hinder viral adhesion to the fish epithelial cells in about 40%. (**B**) In the present study we provide evidence that the binding of Drgal1-L2 to either the IHNV virions or epidermal mucus could hinder viral adhesion to mucus by about 30–60%, suggesting that when the mucus biofilm is continuous and intact as presumably found under normal environmental and physiological conditions, the binding of galectins to mucus glycans can further hinder viral adhesion to the fish surface, in synergy with the galectin bound to the glycosylated viral envelope. (**C**) In the present study we also present evidence that the binding of Drgal1-L2 to epithelial cell surface viral receptor glycans such as fibronectin and β1integrin could also hinder viral adhesion to the epithelial cell surface by about 30–40%, suggesting that under stressful conditions (such as high density aquaculture, and/or environmental stressors such as temperature, pH, or presence of pollutants) in which either by mechanical or physio-pathological loss of continuity of the epidermal mucus film, the binding of galectins to selected glycans on the epithelial cell surface that function as viral receptors, could hinder such virion–receptor interactions and constitutes another level of protection against viral adhesion and infection.

Drgal1-L2 also hindered adhesion of the IHNV virions to the epidermal mucus, either by binding to the virion glycosylated envelope, to the cell surface glycosylated virus receptors, or to the mucus glycans (Figure 13). This observation opens the possibility that Drgal1-L2 present in the epidermal mucus could function as a defense factor by hindering adhesion of IHNV to the epidermal mucus glycans and therefore prevent access of the virions to the cell surface. It has been extensively reported that the mucus film coating mucosal epithelia such as that in the mammalian airway can act both as a mechanical and bioactive antimicrobial barrier by trapping potential pathogens in the mucus matrix aided by the diverse repertoire of antibodies, lectins, and other agglutinating and mucin-binding factors [187,188]. The immobilized bacteria, viruses, and eukaryotic parasites are killed by the numerous antimicrobial peptides, lysozyme, antibodies, lectins, complement, and other effector factors, and are regularly cleared by the ciliary activity of the epithelia that promotes mucus transport along the airway [187,188]. In fish, the mucus film coating the skin, gills, gut and all other mucosal epithelia which like the mammalian epidermal mucus is also rich in antimicrobial factors, is regularly sloughed off clearing all potential pathogens and parasites that may be immobilized in the mucus matrix [51,52,53,54,189]. Some viruses like influenza, SARS-CoV-2, and HPV, however, can take advantage of their immobilization in the mucus matrix, and overcome it through the activity of enzymes or the biophysical properties of their surfaces to access the cell surface receptors for entry into the underlying host epithelial cells [190,191,192,193]. It remains unknown if IHNV is endowed with these or other mechanisms that once in the mucus matrix, enable access of the virions to the cell surface, but it is tempting to speculate that the Drgal1-L2-mediated inhibition of adhesion of the virions to the epidermal mucus film would allow them to be washed away by the water turbulence at the fish interface with the environment during active swimming. If this is the actual mechanism, then it would represent the “safest” strategy of the fish host for preventing infection. The results of this study, however, must be interpreted with great caution, as the use of immortalized fish cell lines and the use of surrogate glycoproteins (fibronectin and β1integrin) impose considerable limitations that will be overcome with the corroboration of the observations in an in vivo system (e.g., zebrafish), implementing loss-of-function approaches targeting the galectin and its ligands on the epithelial cell surface.

## 5. Conclusions

In conclusion, our study strongly suggests that Drgal1-L2 can prevent IHNV infection by alternative or synergistic mechanisms, by either binding to the virion glycosylated envelope, to cell surface receptors such as fibronectin and possibly β1integrin, or to mucus glycans, thereby hindering viral adhesion to the epithelial cell surface or to the epidermal mucus film. Importantly, although supportive of these concepts, our in vitro observations reported herein will be validated by our current in vivo experimental approaches in zebrafish aimed at rigorously testing the proposed hypothesis.

## Figures and Tables

**Table 1:** Bovine fibronectin and recombinant β1integrin expressed in CHO cells are glycosylated and display the galectin ligand Galβ1-4GlcNAc.

Lectin	Fibronectin	β1integrin
SNA	5 *	5
MAA II	3	1
ECA	4	3
MAA I	2	4

* Numerical values represent the relative band intensity in a Western blot in an arbitrary scale from 1 to 5.

## Data Availability

The original contributions presented in this study are included in the article/Supplementary Materials. Further inquiries can be directed to the corresponding author.

## Supplementary Materials

The following supporting information can be downloaded at: https://www.mdpi.com/article/10.3390/biom16060882/s1, Figure S1: SPR analysis of interactions of Drgal1-L2 with purified glycoproteins. The experimental data were fitted using a two-state model. Figure S2: Binding of anti-fibronectin antibodies to immobilized purified fibronectin hinders IHNV adhesion to fibronectin in a dose-response manner. Figure S3: Binding of anti-β1integrin antibodies to immobilized purified β1integrin hinders IHNV adhesion to β1integrin in a dose–response manner. Figure S4: Amino acid sequence alignment of fibronectin from zebrafish, bovine, and rabbit. Figure S5: Amino acid sequence alignment of β1integrin from human, zebrafish, and rabbit.

## Abbreviations

IHNV, infectious hematopoietic necrosis virus; EPC, epithelioma papulosum cyprini epithelial cell line (CRL-2872, ATCC) from fathead minnow (*Pimephales promelas*); ZFL, liver cell line (CRL-2643, ATCC) from zebrafish (*Danio rerio*); Drgal1-L2, *Danio rerio* galectin 1-like isoform 2; rDrgal1-L2, recombinant Drgal1-L2; CRD, carbohydrate recognition domain; RT, room temperature; ASF, asialofetuin; PSM, porcine stomach mucin; BSA, bovine serum albumin; FN, fibronectin; HRP, horseradish peroxidase; ABTS, diammonium 2,2′-azinobis(3-ethylbenzothiazoline-6-sulfonate); BME, 2(β)-mercaptoethanol; PBS, phosphate-buffered saline, (0.01 M Na_2_HPO_4_/0.15 M NaCl/0.01% NaN_3_, pH 7.5); PBS/BME, PBS containing 0.01 M BME; PBS (1:10), PBS diluted 10 fold with water; SDS, sodium dodecyl sulfate; PAGE, polyacrylamide gel electrophoresis; PCR, polymerase chain reaction; ORF, open reading frame; Tris, Tris(hydroxymethyl) aminomethane; LPS, lipopolysaccharide; OPS, O-antigen polysaccharide; PFA, paraformaldehyde; ConA, Concanavalin A; MAA I, *Maackia amurensis* agglutinin I; MAA II, *M. amurensis* agglutinin II; PNA, peanut (*Arachis hypogea*) agglutinin; SBA, soybean (*Glycine max*) agglutinin; SNA, *Sambucus nigra* agglutinin; UEA, *Ulex europaeus* agglutinin; WGA, wheat germ (*Triticum vulgaris*) agglutinin; ECA, *Erythrina cristagalli* agglutinin.
