# Supplementary material for: The Proto Type Galectin Drgal1-L2 from Zebrafish Hinders Infection by the Infectious Hematopoietic Necrosis Virus by Binding to Its Glycosylated Receptors on the Epithelial Cell Surface
Source: Biomolecules. 2026 Jun 15;16(6):882. doi: 10.3390/biom16060882 (PMC13297538; doi:10.3390/biom16060882)
Supplement: Supplementary file 1 [file biomolecules-16-00882-s001.zip › biomolecules-4267794-supplementary.pdf]

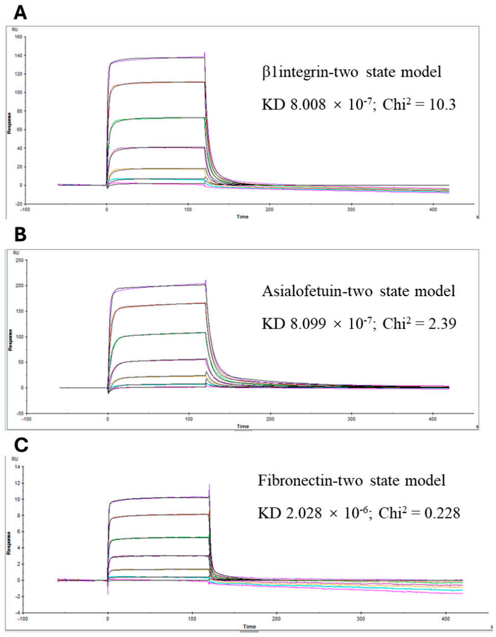

Supplementary Figure S1. SPR analysis of interactions of Drgal1-L2 with purified glycoproteins. The experimental data were fitted using a two-state model.

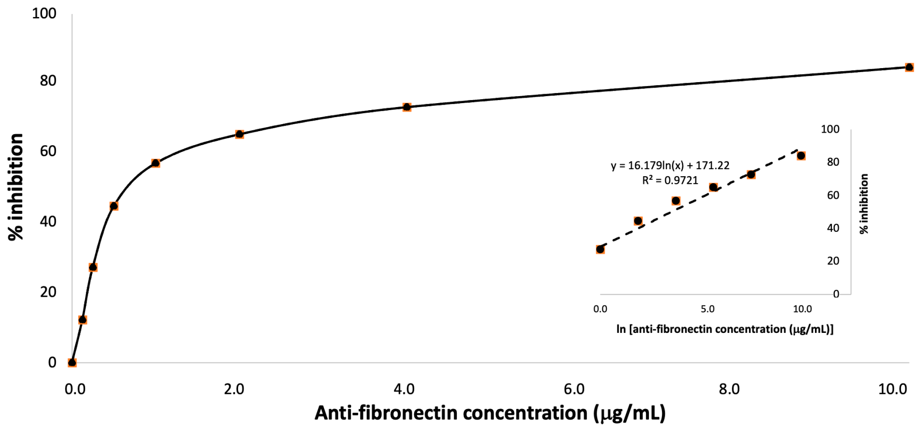

Supplementary Figure S2. Binding of anti-fibronectin antibodies to immobilized purified fibronectin hinders IHNV adhesion to fibronectin in a dose-response manner. Adhesion of IHNV to immobilized purified fibronectin that had been pre-incubated with increasing concentrations of anti- fibronectin antibodies was assessed by ELISA: Fibronectin (5 µg/mL) was immobilized in 96-well microtiter plates and incubated with increasing concentrations of anti-fibronectin antibodies (0-10 µg/mL in PBS), then incubated with biotinylated IHNV (7.5 µg/mL) for 2 h. After washing, IHNV adhesion was assessed with HRP- conjugated streptavidin at (0.15 mg/mL concentration). The percentage of inhibition was calculated over the adhesion without antibody (PBS only) and plotted.

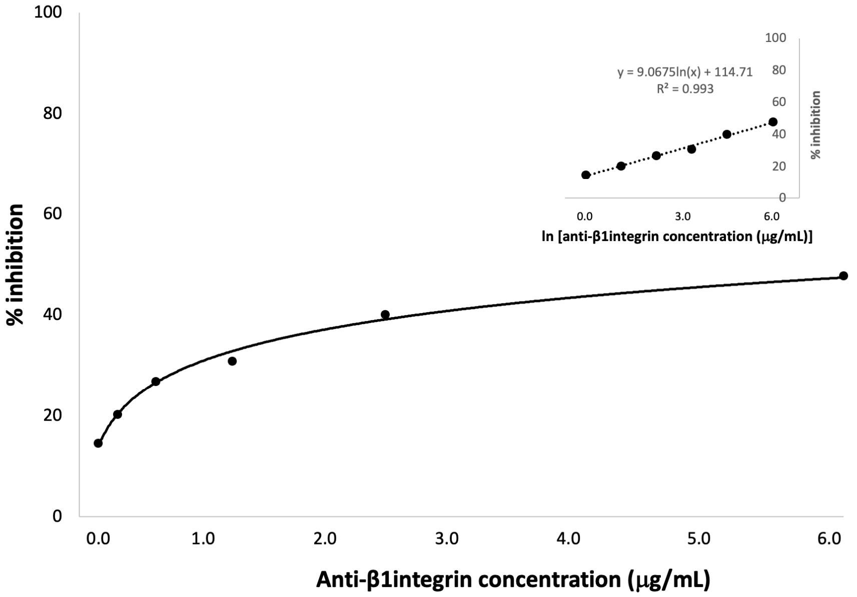

Supplementary Figure S3. Binding of anti-β1integrin antibodies to immobilized purified β1integrin hinders IHNV adhesion to β1integrin in a dose–response manner. Adhesion of IHNV to immobilized β1integrin that had been pre-incubated with increasing concentrations of anti-β1integrin antibodies was assessed by ELISA: β1integrin (5 µg/mL) was immobilized in 96-well microtiter plates and incubated with increasing concentrations of anti-β1integrin antibodies (0-6 µg/mL in PBS), then incubated with biotinylated IHNV (7.5 µg/mL) for 2 h. After washing, IHNV adhesion was assessed with HRP- conjugated streptavidin at (0.15 mg/mL concentration). The percentage of inhibition was calculated over the adhesion without antibody (PBS only) and plotted.

(a) Alignment of fibronectin amino acid sequences from human, rabbit, and zebrafish

```
CLUSTAL O(1.2.4) multiple sequence alignment


AAU14809.1                  MTRE---SVKRLLLLLCIGGSVHCMPQSAGKSKRQAQQQQIHLDTVYEEARSLAIHESGC      57
sp|P07589.4|FINC_BOVIN      MLGGPGPGLLLLLAVLSLGTAVPSA--GASKSRRQAQQIVQ-----PQSPLTVSQSKPGC      53
XP_017198575.1              MLGGPGPGLLL-LSVLCLGTAVPST--AASKSKRQAQQIVQ-----PQSPVAVSQSKPGC      52
                            *      .:   * :*.:* :* .   .*.**:*****         :.  :::  : **

AAU14809.1                  NDNGRVYRMNDEWERPYMDSTLKCTCEG-ASGVKCRSKPAAEESCYDKFNARSYRVGETY      116
sp|P07589.4|FINC_BOVIN      YDNGKHYQINQQWERTYLGSALVCTCYGGSRGFNCESKPEPEETCFDKYTGNTYRVGDTY      113
XP_017198575.1              YDNGKHYQINQQWERTYLGNALVCTCYGGSRGFNCESKPEPEETCFDKYTGNTYRVGDTY      112
                             ***: *::*::*** *:..:* *** * : *.:*.***  **:*:**:...:****:**

AAU14809.1                  ERPKDNMIWDCTCIGSGKGKISCTIANRCHEGGNSYRIGDTWTRPHDTGDYMLECVCLGN      176
sp|P07589.4|FINC_BOVIN      ERPKDSMIWDCTCIGAGRGRISCTIANRCHEGGQSYKIGDTWRRPHETGGYMLECVCLGN      173
XP_017198575.1              ERPKDSMIWDCTCIGAGRGRISCTIANRCHEGGQSYKIGDTWRRPHETGGYMLECVCLGN      172
                            *****.*********:*:*:************:**:***** ***:**.**********

AAU14809.1                  GKGEWTCKPVAERCYDDSLGSSYVVGQTWQKPYQGWMIVDCTCLGEGNGRITCTSRNRCN      236
sp|P07589.4|FINC_BOVIN      GKGEWTCKPIAEKCFDQAAGTSYVVGETWEKPYQGWMMVDCTCLGEGSGRITCTSRNRCN      233
XP_017198575.1              GKGEWTCKPIAEKCFDHAAGTSYVVGETWEKPYQGWMMVDCTCLGEGSGRITCTSRNRCN      232
                            *********:**:*:*.: *:*****:**:*******:*********.************

AAU14809.1                  DQDTRTSYRIGETWSKIDSSGHTLQCLCTGNGRGEWKCDRHAASHVIPAIGTGSTVTHRV      296
sp|P07589.4|FINC_BOVIN      DQDTRTSYRIGDTWSKKDNRGNLLQCICTGNGRGEWKCERHTSLQTTS-AGSGS--FTDV      290
XP_017198575.1              DQDTRTSYRIGDTWSKKDNRGNLLQCICTGNGRGEWKCERHASLQTTS-TGSGP--FSDV      289
                            ***********:**** *. *: ***:***********:**:: :.    *:*      *

AAU14809.1                  TPVMNQVNVLNELIEEGNCKTDSGVSYYNNMSWIRTQGTKEMLCTCVGGGISCEEQDGQS      356
sp|P07589.4|FINC_BOVIN      RTAIYQPQPHPQPPPYGHCVTDSGVVYSVGMQWLKTQGNKQMLCTCLGNGVSCQET-AVT      349
XP_017198575.1              RAAVYQPQPHPQPAPYGHCVTDSGVVYSEGMQWLKTQGNKQMLCTCLGNGVSCQET-AVT      348
                              .: * :   :    *:* ***** *  .*.*::***.*:*****:*.*:**:*  . :

AAU14809.1                  QVYGGNSGGQPCVFPFVFSGNTHYSCISEGRSDGQLWCSTTSDYDSDGLYSFCTQKN-LL      415
sp|P07589.4|FINC_BOVIN      QTYGGNSNGEPCVLPFTYNGKTFYSCTTEGRQDGHLWCSTTSNYEQDQKYSFCTDHT-VL      408
XP_017198575.1              QTYGGNSNGEPCVLPFTYNGRTFYSCTTEGRQDGHLWCSTTSNYEQDQKYSFCTDHTAVL      408
                            *.*****.*:***:**.:.*.*.*** :***.**:*******:*:.*  *****::. :*

AAU14809.1                  VTTRGGNSNGALCQFPFKYNGRNYTDCTAEGRRDGMKWCGTTADNDREQKYGFCPMAAHE      475
sp|P07589.4|FINC_BOVIN      VQTRGGNSNGALCHFPFLYNNHNYTDCTSEGRRDNMKWCGTTQNYDADQKFGFCPMAAHE      468
XP_017198575.1              VQTRGGNSNGALCHFPFLYNNHNYTDCTSEGRRDNMKWCGTTQNYDADQKFGFCPMAAHE      468
                            * ***********:*** **.:******:*****.******* : * :**:*********
```

(b) Alignment of fibronectin amino acid sequences from human, rabbit, and zebrafish

```
AAU14809.1                  EVCTVN-DVMYRVGDEWDKRHDTLGHMMRCTCQGNGRGEWNCISHTQLKDQCVVNGQTYD      534
sp|P07589.4|FINC_BOVIN      EICTTNEGVMYRIGDQWDKQHD-MGHMMRCTCVGNGRGEWTCVAYSQLRDQCIVDGITYN      527
XP_017198575.1              EICTTNEGVMYRIGDQWDKQHD-MGHMMRCTCVGNGRGEWTCVAYSQLRDQCIVDDITYN      527
                            *:**.* .****:**:***:** :******** *******.*:::**:***:*:. **:

AAU14809.1                  VNETFDKRHDQGYMMNCTCFGQGRGRWKCDAIDQCQEPETKVFYQIGQTWNKVIQGTPYR      594
sp|P07589.4|FINC_BOVIN      VNDTFHKRHEEGHMLNCTCFGQGRGRWKCDPVDQCQDSETRTFYQIGDSWEKYLQGVRYQ      587
XP_017198575.1              VNDTFHKRHEEGHMLNCTCFGQGRGRWKCDPVDQCQDSETRTFYQIGDSWEKYVHGVRYQ      587
                            **:**.***::*:*:*************** :****: **:.*****::*:* ::*. *:

AAU14809.1                  CSCYGNGIGEMACEPLQS----TAPVRLIITEAGNQPNSHPIQWNAPPSAHITQYILKWR      650
sp|P07589.4|FINC_BOVIN      CYCYGRGIGEWACQPLQTYPDTSGPVQVIITETPSQPNSHPIQWSAPESSHISKYILRWK      647
XP_017198575.1              CYCYGRGIGEWHCQPLQTYPGTSGPVQVIITETPSQPNSHPIQWNAPEPSHISKYILRWR      647
                            * ***.****  *:***:    :.**::****: .*********.** .:**::***:*:

AAU14809.1                  VKNTRTPWKEVTIPGHINSYTISGLKAGLTYEGQLISILRYGPREVTRFDFTTTYGSLA-      709
sp|P07589.4|FINC_BOVIN      PKNSPDRWKEATIPGHLNSYTIKGLRPGVVYEGQLISVQHYGQREVTRFDFTTTSTSPAV      707
XP_017198575.1              PKNSVGRWKEATIPGHLNSYTIKGLRPGVMYEGQLISIQQYGHREVTRFDFTTTSTSTPV      707
                             **:   ***.*****:*****.**:.*: *******: :** **********  *

AAU14809.1                  ---KAEGETTQPRQVVDTSESVTEITSNSFVISWVSASDTVSGFRVEYELSEEGAQKRVI      766
sp|P07589.4|FINC_BOVIN      TSNTVTGETTPLSPVVATSESVTEITASSFVVSWVSASDTVSGFRVEYELSEEGDEPQYL      767
XP_017198575.1              TSNTVTGETTPLSPVVATSESVTEITASSFVVSWVSASDTVSGFLVEYELSEEGDEPKYL      767
                               .. ****    ** *********:.***:************ ********* : : :

AAU14809.1                  DLPRTTTSVNIQDLLPGRRYNVDVYEVNPEGDTKLILTTTQTTAPDAPTNHEVSNVQETS      826
sp|P07589.4|FINC_BOVIN      DLPSTATSVNIPDLLPGRKYTVNVYEISEEGEQNLILSTSQTTAPDAPPDPTVDQVDDTS      827
XP_017198575.1              DLPSTVTSVNIPDLLPGRKYIVNVYQISEEGKQSLILSTSQTTAPDAPPDPTVDQVDDTS      827
                            *** *.***** ******:* *:**::. **. .***:*:********  :  *.:*::**

AAU14809.1                  IVIRWSKPKAPITGYRVVYTPSVEGSSTELILPETQTSVTLGDLRPGLSYNISIYSVEDN      886
sp|P07589.4|FINC_BOVIN      IVVRWSRPRAPITGYRIVYSPSVEGSSTELNLPETANSVTLSDLQPGVQYNITIYAVEEN      887
XP_017198575.1              IVVRWSRPQAPITGYRVVYSPSVEGSSTELNLPETANSVTLSDLQPGVQYNITIYAVEEN      887
                            **:***:*:*******:**:********** **** .****.**:**:.***:**:**:*

AAU14809.1                  LESEPLVLQASTAGEQQPEEIQAPTDLQFYEVTDVKITITWTGPPNEVSGYRVTFAPVST      946
sp|P07589.4|FINC_BOVIN      QESTPVFIQQETTGVPRSDKVPPPRDLQFVEVTDVKITIMWTPPESPVTGYRVDVIPVNL      947
XP_017198575.1              QESAPVFIQQETTGVPRSDEVPPPKDLQFVEVTDVKVTIMWTPPDSAVTGYRVDVLPVHL      947
                             ** *:.:* .*:*  : :::  * **** ******:** ** *  * . *:**** . **

AAU14809.1                  DGQAQRPLQLPVTHNAYAELMRLQPGTLYRFHIYAVSGGVESEPLIGEKSTKPDAPTDLR      1006
sp|P07589.4|FINC_BOVIN      PGEHG--QRLPVSRNTFAEVTGLSPGVTYHFKVFAVNQGRESKPLTAQQATKLDAPTNLQ      1005
XP_017198575.1              PGENG--QRLPVSRNTFAEITGLSPGVTYYFKVFAVNHGRESRPLTAQQATKLDAPTNLQ      1005
```

(c) Alignment of fibronectin amino acid sequences from human, rabbit, and zebrafish

```
AAU14809.1                  FTDITDDSALVIWSFPRAQVTGYRLFISIGS-SSPKQLRIPGHKSQYKLPNLQPDTEYRV      1065
sp|P07589.4|FINC_BOVIN      FINETDTTVIVTWTPPRARIVGYRLTVGLTRGGQPKQYNVGPAASQYPLRNLQPGSEYAV      1065
XP_017198575.1              FVNETDSSVLVIWTPPRARITGYQLTIGPTRGGQPKQHNVGPTVSKYLLRNLQPGSEYTV      1065
                            * : ** :.:* *: ***::.**:* :.    ..*** .:    *:* * ****.:** *

AAU14809.1                  TLHSEQGNTLSEGITDTFRTTQPTGNAPRFNTEVTDTAIIVTWIPVAKFSYRMSVKPSQS      1125
sp|P07589.4|FINC_BOVIN      SLVAVKGNQQSPRVTGVFTTLQPLGSIPHYNTEVTETTIVITWTPAPRIGFKLGVRPSQG      1125
XP_017198575.1              TLIAVKGNNQSPKVTGVFTTLQPQSSIPPYSTEVTETSIVITWTPAPRIGFKLGVRPSQG      1125
                            :* : :**  *  :*..* * ** .. * :.****:*:*::** *. ::.:::.*:***.

AAU14809.1                  GETPREETSGSGRIYISGLTPGLEYIYGLQPLFSGRKHGSPITNKVVTSLSPPTDLNVEP      1185
sp|P07589.4|FINC_BOVIN      GEAPREVTSESGSIVVSGLTPGVEYVYTISVLRDGQERDAPIVKKVVTPLSPPTNLHLEA      1185
XP_017198575.1              GEAPREVTSESGSIVVSGLTPGVEYVYSIQVLRDGKERDAPIVNTVVTPLSPPTNLHLEA      1185
                            **:*** ** ** * :******:**:* :. * .*:::.:**.:.*** *****:*::*

AAU14809.1                  NSVNGELNVRWRGTKSPDITGYRVTGTPINGQRGVSLEESVRGDETSCILENLSPGVDYN      1245
sp|P07589.4|FINC_BOVIN      NPDTGVLTVSWERSTTPDITGYRITTTPTNGQQGYSLEEVVHADQSSCTFENLSPGLEYN      1245
XP_017198575.1              NPDTGVLTVSWEKSTTPDITGYRITTTPTNGQQGYSLEEVVHADQNSCIFENLSPGLEYN      1245
                            *  .* *.* *. :.:*******:* ** ***:* **** *:.*:.** :******::**

AAU14809.1                  ISVYTVKNHLESEPISTSVTQDVPKVGDLSFVDFTDTTIGIRWTPLNYQAVTGYHITVVT      1305
sp|P07589.4|FINC_BOVIN      VSVYTVKDDKESVPISDTIIPEVPQLTDLSFVDITDSSIGLRWTPLNSSTIIGYRITVVA      1305
XP_017198575.1              VSVYTVKDDKESVPVSDTIIPEVPQLTDLSFVDITDSSIGLRWTPLNSSTIIGYRITVVA      1305
                            :******:. ** *:* ::  :**:: ******:**::**:****** .:: **:****:

AAU14809.1                  SGQNFPILEDVVNSSVNYYTIRGLEPGINYDISVSTVTDEAESVPSVITQQTQTAVPAPT      1365
sp|P07589.4|FINC_BOVIN      AGEGIPIFEDFVDSSVGYYTVTGLEPGIDYDISVITLINGGESAPTTLTQQ--TAVPPPT      1363
XP_017198575.1              AGEGIPIFEDFVDSSVGYYTVTGLEPGIDYDISVITLINGGESAPTTLTQQ--TAVPSPT      1363
                            :*:.:**:**.*:***.***: ******:***** *: : .**.*:.:***  **** **

AAU14809.1                  NLYISEVGADSMHVSWTAPSVQPSEISRFVIRYHPTNNDDDTQEVNVGGGTTSFVLQNLL      1425
sp|P07589.4|FINC_BOVIN      DLRFTNVGPDTMRVTWAPPSS--IELTNLLVRYSPVKNEEDVAELSISPSDNAVVLTNLL      1421
XP_017198575.1              DLRFTNIGPDTMRVTWAPPPS--IELTNFLVRYSPVKNEEDVAELSISPSDNAVVLTNLL      1421
                            :* ::::* *:*:*:*: *     *::.:::** *.:*::*. *:.:. . .:.** ***

AAU14809.1                  PNTEYLVKVVCVYDDRESEPVTGIQKTKLDSPTNLDFSDVSTNSFTVHWMAPRAVITGYR      1485
sp|P07589.4|FINC_BOVIN      PGTEYLVSVSSVYEQHESIPLRGRQKTALDSPSGIDFSDITANSFTVHWIAPRATITGYR      1481
XP_017198575.1              PGTEYLVSVSSVYEQHESTPVRGRQKTGLDAPTGIDFSDVTPNSFTVYWTPPRAPITGFW      1481
                            *.*****.* .**:::** *: * *** **:*:.:****:: *****:*  *** ***:

AAU14809.1                  LRYQPTS-GGRAKDERLPPTRNYFTLVNLAPETEYTVHIYAVSSNIESLPLTGTQATVSD      1544
sp|P07589.4|FINC_BOVIN      IRHHPENMGGRPREDRVPPSRNSITLTNLNPGTEYVVSIVALNSKEESLPLVGQQSTVSD      1541
XP_017198575.1              IRHHPEHGVGRPREDRVPPSRNSITLTNLNPGTEYVVSIVALNGREQSPPLIGQQSTVSD      1541
                            :*::*    ** :::*:**:** :**.** * ***.* * *:.*. *:... :* ** * *:****
```

Supplementary Figure S4. Amino acid sequence alignment of fibronectin from zebrafish, bovine, and rabbit: The amino acid sequences of fibronectin from zebrafish (AAU14809.1), bovine (spP07589.4), and rabbit (XP_017198575.1) were obtained from the NCBI public database and aligned.

(a) Alignment of β1-integrin amino acid sequences from human, rabbit, and zebrafish

```
CLUSTAL O(1.2.4) multiple sequence alignment

ADR79324.1          MDVRLLLISVLLG---LSRAQQDGNECTKASAQSCGECIQAGEKCGWCTDEGFLKQGEQK      57
AAH20057.1          MNLQPIFWIGLISSVCCVFAQTDENRCLKANAKSCGECIQAGPNCGWCTNSTFLQEGMPT      60
XP_008272963.1      MNLQLIFWIGLLGSICCVFGQTDENRCLKANAKSCGECIQAGPNCGWCTNSTFLQEGMPT      60
                    *::: ::   *:.      .* * *.* **.*:********* :*****:. **::*  .

ADR79324.1          STRCDEIEALEKKGCSKASIENPRGKITIVKNQPVTNRTKN-GAKLKPDQITQIQPQQLS      116
AAH20057.1          SARCDDLEALKKKGCPPDDIENPRGSKDIKKNKNVTNRSKGTAEKLKPEDITQIQPQQLV      120
XP_008272963.1      SARCDDLEALKKKGCHPDDIENPRGSRDIKKNKNVTNRSKGTAEKLQPEDITQIQPQQLV      120
                    *:***::***:****    .******.  * **: ****:*. . **:*::*********

ADR79324.1          LNLRSGEAQKFTLKFKRAEDYPIDLYYLMDLSYSMKDDLENVKNLGTDLMKEMQKITSDF      176
AAH20057.1          LRLRSGEPQTFTLKFKRAEDYPIDLYYLMDLSYSMKDDLENVKSLGTDLMNEMRRITSDF      180
XP_008272963.1      LQLRSGEPQTFTLKFKRAEDYPIDLYYLMDLSYSMKDDLENVKSLGTDLMNEMRRVTSDF      180
                    *.***** *.*********************************.******:**:::****

ADR79324.1          RIGFGSFVEKTVMPYISTTPAKLLNPCTSDQNCTSPFSYKNVLSLTDDGSQFNSLVSRQQ      236
AAH20057.1          RIGFGSFVEKTVMPYISTTPAKLRNPCTSEQNCTSPFSYKNVLSLTNKGEVFNELVGKQR      240
XP_008272963.1      RIGFGSFVEKTVMPYISTTPAKLKNPCTSEQNCTSPFSYKNVLSLTDRGEEFNELVGKQR      240
                    *********************** *****:****************: *. **.**.:*:

ADR79324.1          ISGNLDSPEGGFDAIMQVAVCGNQIGWRNVTRLLVFSTDAGFHFAGDGKLGGIVLPNDGK      296
AAH20057.1          ISGNLDSPEGGFDAIMQVAVCGSLIGWRNVTRLLVFSTDAGFHFAGDGKLGGIVLPNDGQ      300
XP_008272963.1      ISGNLDSPEGGFDAIMQVAVCGSLIGWRNVTRLLVFSTDAGFHFAGDGKLGGIVLPNDGQ      300
                    **********************. ************************************:

ADR79324.1          CHLQDNIYTMSHYYDYPSIAHLVQKLSENNIQTIFAVTEEFQPVYQELKNLIPKSAVGTL      356
AAH20057.1          CHLENNMYTMSHYYDYPSIAHLVQKLSENNIQTIFAVTEEFQPVYKELKNLIPKSAVGTL      360
XP_008272963.1      CHLEHNEYTMSHYYDYPSIAHLVQKLSENNIQTIFAVTEEFQPVYKELKNLIPKSAVGTL      360
                    ***:.* *************************************:**************

ADR79324.1          TSDSNNVIKLIIDAYNSLSSEVILENSKLPEGVSISYVSHCKNGVSGTGDTGRKCSNISI      416
AAH20057.1          SANSSNVIQLIIDAYNSLSSEVILENGKLSEGVTISYKSYCKNGVNGTGENGRKCSNISI      420
XP_008272963.1      SANSSNVIQLIIDAYNSLSSEVILENSKLPEGVTINYKSYCKNGVNGTGENGRKCSNISI      420
                    :::*.***:****************.** ***:*.* *:*****.***:.*********

ADR79324.1          GDEVAFEVAITAKGCPLNGKSETMKIKLLGFTEEVEVVLNFICECECHKDGIKNSPVCHF      476
AAH20057.1          GDEVQFEISITSNKCPKK-DSDSFKIRPLGFTEEVEVILQYICECECQSEGIPESPKCHE      479
XP_008272963.1      GDEVHFEISITSNQCPVK-HSETIKIKPLGFTEEVEVVLEFICKCECQDTGIPRSPQCHD      479
                    **** **::**:: ** : .*:::**: *********:*::**:***:. ** .** **
```

(b) Alignment of β1-integrin amino acid sequences from human, rabbit, and zebrafish

```
ADR79324.1          GNGTLECGACRCNEGRIGRVCECSKDEVRTEDLDANCRMDNGTDICSNNGDCVCGTCECK        536
AAH20057.1          GNGTFECGACRCNEGRVGRHCECSTDEVNSEDMDAYCRKENSSEICSNNGECVCGQCVCR        539
XP_008272963.1      GNGTFECGACRCNEGRVGRHCECSTDEVNSEDMDAYCRKENSSEICSNNGECVCGQCVCR        539
                    ****:***********:** ****.***.:**:** ** :*.::******:**** * *:

ADR79324.1          KRDNPEERYSGKFCECDNFNCDRSNNKLCGGHGRCDCRKCICDANYTGSACDCSLDTSTC        596
AAH20057.1          KRDNTNEIYSGKFCECDNFNCDRSNGLICGGNGVCKCRVCECNPNYTGSACDCSLDTSTC        599
XP_008272963.1      KRDNTNEIYSGKFCECDNFNCDRSNGLICGGNGVCKCRVCECNPNYTGSACDCSLDTTSC        599
                    **** :* *****************. :***:* *.** * *: *************::*

ADR79324.1          LASNKQICNGRGNCECGACKCTDTKFQGPTCEICPTCPGVCTEHKDCVQCRAFGTGDKKD        656
AAH20057.1          EASNGQICNGRGICECGVCKCTDPKFQGQTCEMCQTCLGVCAEHKECVQCRAFNKGEKKD        659
XP_008272963.1      MATNGQICNGRGICECGVCKCTDPKFQGQTCEMCQTCLGVCAEHKECVQCRAFNKGEKKD        659
                     *:* ******* ****.***** **** ***:* ** ***:***:*******..*:***

ADR79324.1          TCEEQCSYFTMKVVKKKEDLPQPNDQPIINHCKERDANDCWFFFTYATRNDSSVMVHVAE        716
AAH20057.1          TCTQECSYFNITKVESRDKLPQPVQPDPVSHCKEKDVDDCWFYFTYSVNGNNEVMVHVVE        719
XP_008272963.1      KCAQECSHFNITKVENRDKLPQPGQVDPLSHCKEKDVDDCWFYFTYSVNGNNEAIVHVVE        719
                    .* ::**:*.:. *:.::.**** :   :.****:*.:****:***:...:...:***.*

ADR79324.1          ELECPSGPDIIPIVAGVVAGIVLIGLALLLIWKLLMIIHDRREFAKFEKEKMNAKWDAGE        776
AAH20057.1          NPECPTGPDIIPIVAGVVAGIVLIGLALLLIWKLLMIIHDRREFAKFEKEKMNAKWDTGE        779
XP_008272963.1      TPECPTGPDIIPIVAGVVAGIVLIGLALLLIWKLLMIIHDRREFAKFEKEKMNAKWDTQE        779
                      ***:***************************************************: *

ADR79324.1          NPIYKSAVTTVVNPKYEGK--       795
AAH20057.1          NPIYKSAVTTVVNPKYEGK--       798
XP_008272963.1      NPIYKSPINNFKNPNYGRRAG       800
                    ****** :... **:*  :
```

Supplementary Figure S5. Amino acid sequence alignment of β1integrin from human, zebrafish, and rabbit: The amino acid sequences of CD147 from human (ADR79324.1), zebrafish (AAH20057.1), and rabbit (XP_008272963.1) were obtained from the NCBI public database and aligned.
